# An FSV analysis approach to verify the robustness of the triple-correlation analysis theoretical framework

**DOI:** 10.1038/s41598-023-35900-3

**Published:** 2023-06-14

**Authors:** Robert M. X. Wu, Zhongwu Zhang, Huan Zhang, Yongwen Wang, Niusha Shafiabady, Wanjun Yan, Jinwen Gou, Ergun Gide, Siqing Zhang

**Affiliations:** 1 Faculty of Engineering and Information Technology, University of Technology Sydney, Sydney, 2000 Australia; 2 School of Geography, Shanxi Normal University, Taiyuan, 030009 China; 3 Shanxi Fenxi Mining Industry (Group) Co. Ltd, Jiexiu, 032000 China; 4 Faculty of Science and Technology, Charles Darwin University (Sydney Campus), Sydney, 2000 Australia; 5 Shanxi Fenxi Mining Zhongxing Coal Industry Co. Ltd, Lvliang, 030500 China; 6 School of Engineering and Technology, Central Queensland University, Sydney, 2000 Australia; 7 Taiyuan Normal University, Taiyuan, 030009 China

**Keywords:** Environmental sciences, Natural hazards

## Abstract

Among all the gas disasters, gas concentration exceeding the threshold limit value (TLV) has been the leading cause of accidents. However, most systems still focus on exploring the methods and framework for avoiding reaching or exceeding TLV of the gas concentration from viewpoints of impacts on geological conditions and coal mining working-face elements. The previous study developed a Trip-Correlation Analysis Theoretical Framework and found strong correlations between gas and gas, gas and temperature, and gas and wind in the gas monitoring system. However, this framework's effectiveness must be examined to determine whether it might be adopted in other coal mine cases. This research aims to explore a proposed verification analysis approach—First-round—Second-round—Verification round (FSV) analysis approach to verify the robustness of the Trip-Correlation Analysis Theoretical Framework for developing a gas warning system. A mixed qualitative and quantitative research methodology is adopted, including a case study and correlational research. The results verify the robustness of the Triple-Correlation Analysis Theoretical Framework. The outcomes imply that this framework is potentially valuable for developing other warning systems. The proposed FSV approach can also be used to explore data patterns insightfully and offer new perspectives to develop warning systems for different industry applications.

## Introduction

As the world’s largest coal producer, China’s coal mine industry accounted for about 46% of global coal production in 2020^1,2^. Gas accidents are severe that must be addressed by coal mining industry managers in China^3^. Among all the gas disasters, gas concentration exceeding the threshold limit value (TLV) has been the leading cause of accidents^4^. Therefore, gas monitoring systems for real-time TLV have been adopted in China’s coal mines. However, most systems still focus on exploring the methods and framework for avoiding reaching or exceeding TLV of the gas concentration from viewpoints of impacts on geological conditions and coal mining working-face elements. When the gas data outputs reach or exceed TLV, the gas monitoring system alerts the mine’s safety response team^5^.

Up-to-date literature indicates that current studies mainly focus on using machine learning (ML) (including deep learning) approaches to explore warnings or predict models for avoiding exceeding the TLV of the gas concentration. However, a comprehensive literature review in the previous work appears to have at least three significant limitations on using ML methods to predict gas emissions and gas concentrations in the current coal monitoring systems model^5,6^. They include poor (dataset) inputs resulting in inadequate outputs, inaccurate interpreted prediction results, and high cost of the computing hardware for improving the efficiency and effectiveness of the ML models^5^. No published paper fully reports on systems that utilize the collected coal mine data; no attempt has been made to uncover the correlation between gas concentration and other data and apply them to predict gas concentration^4^. Therefore, a previous study developed a Trip-Correlation Analysis Theoretical Framework for developing an innovative integrated gas warning system that indicated significant relationships between gas and gas, gas and temperature, and gas and wind^5^. However, there is a need to examine the effectiveness of the Trip-Correlation Analysis Theoretical Framework, which might be adopted in other coal mine cases.

This research aims to explore a proposed verification analysis approach—First-round—Second-round—Verification round (FSV) analysis approach to verify the robustness of the Trip-Correlation Analysis Theoretical Framework for developing a gas warning system. A mixed qualitative and quantitative research methodology is adopted, including a case study and correlational research. The following sections focus on data sources, methods, results, discussion, conclusion, and data availability.

## Data sources

**Figure 1:**
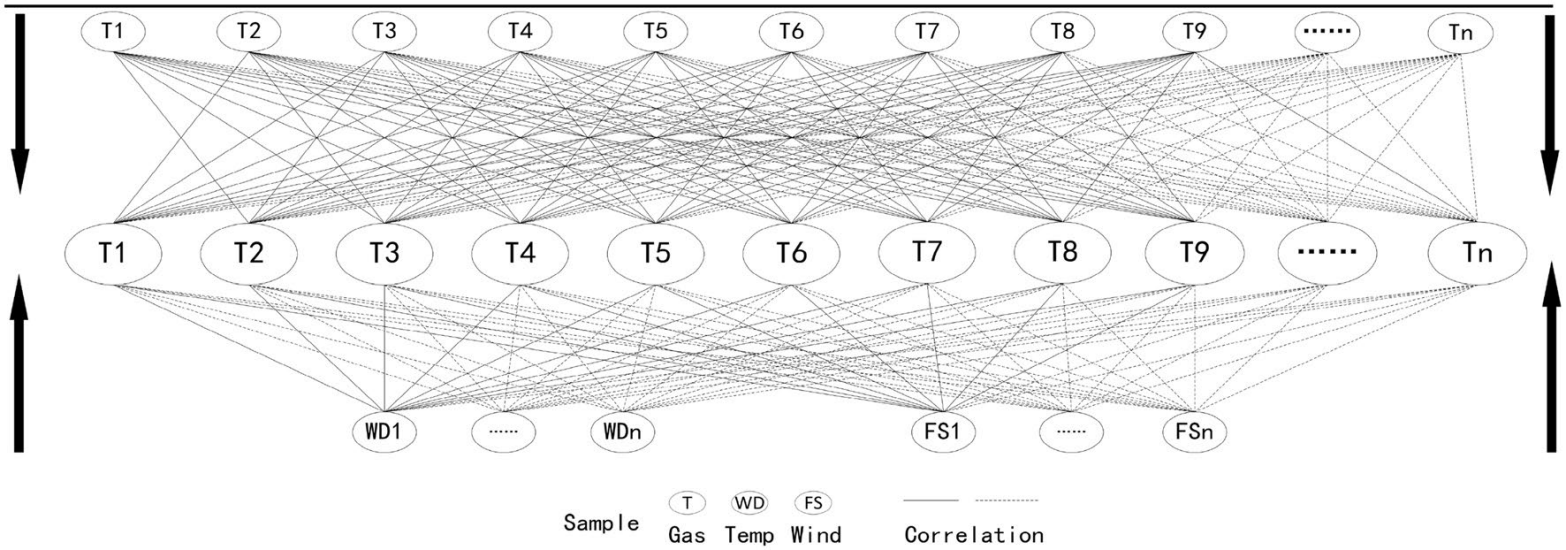
Shown is a Triple-Correlation Analysis Theoretical Framework adopted in this research to propose a research framework comprising the correlation analyses between the gas (from T1 to Tn) and gas (from T1 and Tn), gas (from T1 and Tn) and temperature (from WD1 to WD16), and gas (from T1 to Tn) and wind (from FS1 to FSn).

The previous study found strong correlations between gas and gas, gas and temperature, and gas and wind, which was adopted to develop a Trip-Correlation Analysis Theoretical Framework^5^. It consists of three correlation analyses, including correlation analysis between gas and gas, gas and temperature, and gas and wind (see Fig. 1).

**Figure 2:**
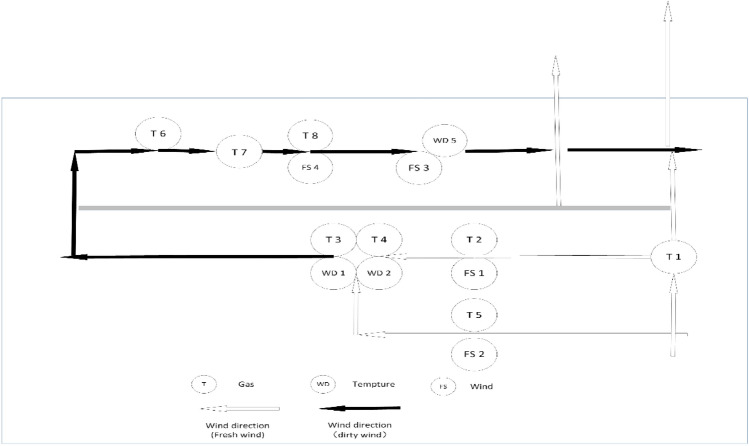
Shown are sensors allocated in the layout map of working-face No.3209 in the Case Study mine, including fourteen sensors -eight gas sensors (from T1 to T8), two temperature sensors (from WD 1 to WD2), and four wind sensors (from FS1 to FS4).

This research is conducted to verify the robustness of the Trip-Correlation Analysis Theoretical Framework. Research data are collected from fourteen sensors, including eight gas sensors (from T1 to T8), two temperature sensors (from WD 1 to WD2), and four wind sensors (from FS1 to FS4) (see Fig. 2).

The gas, temperature, and wind sensor codes can be seen in Tables 1, 2 and 3, respectively.

**Table 1:** Shown are T1 to T8 numbered to each gas sensor and their device code given in the gas monitoring system deployed in the Case Study mine.

No	Gas sensor name	Code	No	Gas sensor name	Code
T1	3209Pre-pumping lane return air T	T060101	T5	3209Protective layer transport alley sub-airway T	T060202
T2	3209Pre-pumping Lane Coal Storage T	T060102	T6	3209Protected layer transport road working face T	T050203
T3	3209Pre-pumping lane split air outlet T	T060103	T7	3209Protective layer transport alley return wind T	T050204
T4	3209Pre-drawing lane working face T	T060104	T8	3209Protected layer transport alley drilling rig T	T050205

**Table 2:** Shown are WD1 and WD2 numbered to each temperature sensor and its device code given in the gas monitoring system deployed in the Case Study mine.

No	Temperature sensor name	Code
WD1	3209Pre-pumping lane return air WD	WD060101
WD2	3209Protected transport lane return air WD	WD060201

**Table 3:** Shown are FS1 to FS4 numbered to each temperature sensor and its device code given in the gas monitoring system deployed in the Case Study mine.

No	Wind sensor name	Code
FS1	3209Pre-pumping lane return air FS	FS060101
FS2	3209pre-pumping lane split air outlet bi-directional FS	FS060102
FS3	3209Protected Transport Lane Return Air FS	FS060201
FS4	3209Protective Layer Transport Lane Split Air Outlet Two-way FS	FS060202

Data are obtained from each sensor for two days between 00:00:00 am on 5 February 2022 and 23:59:00 on 6 February 2022. 5760 data points are initially recorded from each sensor because data collection occurs at 15 s sampling intervals. Thus, 80,640 data points are collected in total, including gas sensors (46,080), temperature sensors (11,520), and wind sensors (23,040). The time series of the dataset outputs of all sensors (gas, temperature, and wind) on 5 February can be seen in Online Appendices 2–4. The time series of the dataset outputs of all sensors (gas, temperature, and wind) on 6 February can be seen in Online Appendices 5–7.

## Methods

A mixed qualitative and quantitative research methodology is adopted, including a case study and correlational research. This research comprises five processes: data acquisition, pre-processing, data analysis, verification, and correlation analysis (see Online Appendix 1). This project adopts a mixed-analysis method for verifying data analysis—FSV.

### Data acquisition

Data are obtained from the Case Study mine—Shanxi Fenxi Mining ZhongXing Coal Industry Co. Ltd (ZhongXing)—a large coal mining company in China.

### Data pre-processing

Data pre-processing is necessary before data analysis since the raw data gathered in most industrial processes usually come with many dataset issues, such as out-of-range values, outliers, missing values, etc^7^. This research performs three data cleaning procedures during pre-processing: eliminating extreme values, outliers, and data standardizing.

Extreme data values (also called extreme values in this paper) are considered the out-of-range values in this research. The extreme values could lead to substantially biased inference and be omitted^8^. Other data quality issues—such as errors in measurement, noise, missing values, etc.—might be impacted by hardware relocation, sensor removal, added detectors, and/or not in-used sensors. Such issues are not discussed in this research. But they will be investigated in further studies.

Outliers come from out-of-order distributions for most datasets. They could substantially influence most parametric tests, which would profoundly impact the statistical analysis and often lead to distortion and possibly inaccurate and erroneous conclusions^9^. Anomaly data were mainly observed as the outliers were presumed to come from a different distribution within most datasets^9^. Anomaly data are considered outliers in this research. The Box-plot technique is used to eliminate extreme values and outliers. The box-plot approach uses the median, the approximate quartiles, and the lowest and highest data points to convey the level, spread, and symmetry of a distribution of data values; this approach could easily be refined to identify outlier data points^10^.

Data standardization is followed as data are collected from the different sensors with various measurements. The most common methods for standardizing data include z-score normalization, min–max standardization, distance-to-target normalization, and raking ranking normalization^11,12^. The Z-score normalization method is used in this research (see Eq. 1)^13^ and computed by SPSS Statistics version 26.

1 $$\mathrm{z}=\frac{(\mathrm{x}-\upmu )}{\upsigma }$$

where z is the standard score, x is the value of the original data, μ Is the average of the dataset, and σ Is the standard deviation of the dataset.

### Two-round data analyses

Two-round data analyses are conducted by using different datasets. The obtained data's reliability and validity should separately be achieved between gas and gas, gas and temperature, and gas and wind.

Several statistical significance levels have been accepted for hypothesis testing, including 0.05, 0.01, and 0.001 in social science studies^14^. *P* values of 0.05 have been considered acceptable for ‘significance’ to determine whether to reject the null hypothesis^15^. However, the smaller the significance value, the lower the risk of rejecting the null hypothesis when it is true; this needs to be balanced by the risk of accepting the null hypothesis when it is not true^16^. Recent research believes a *p* value of 0.01 is often considered highly significant^17^. This research verifies the value of 0.01 is a suitable cut-off for the significance level to lower the risk of rejecting the null hypothesis in developing a gas warning system.

Cronbach’s Alpha confirms the data reliability. If the values of Cronbach’s Alpha were above 0.6, it would be considered fair or above reliability. Exploratory factor analyses confirm the validity analysis. If Kaiser–Meyer–Olkin (KMO) value was greater than 0.6, it would be supposed to be acceptable or above measures. Bartlett’s test of Sphericity should be 0.000 (*p* < 0.001). All average communality values should be greater than 0.6. All of the Anti-image correlations are required to be more than 0.5.

Correlational research is then conducted to indicate that two variables are influenced by a common underlying mechanism^18^. The Pearson correlation analysis method is used for this research. The correlation coefficient is used to evaluate and measure the correlation between pairs of input and output variables. The correlation coefficient’s magnitude indicates that the strength of the relationship depends on how close the coefficient is to − 1 or 1, which is the correlation coefficient range^19^. The following mathematical formulas are used for calculating the Pearson correlation coefficient (see Eq. 2)^20^ and computed by SPSS Statistics version 26.

2 $$\mathrm{r}=\frac{\sum ({\mathrm{x}}_{\mathrm{i}}-\overline{\mathrm{x}})({\mathrm{y}}_{\mathrm{i}}-\overline{\mathrm{y}})}{\sqrt{\sum {({\mathrm{x}}_{\mathrm{i}}-\overline{\mathrm{x}})}^{2}\sum {({\mathrm{y}}_{\mathrm{i}}-\overline{\mathrm{y}})}^{2}}}$$

r will be estimated from (${\mathrm{x}}_{\mathrm{i}}$, ${\mathrm{y}}_{\mathrm{i}}$), the mean value of standard scores of sample points, and the expression equivalent to the above formula is obtained.

Where $\mathrm{r}$ is the correlation coefficient. ${x}_{i}$ is the value of the x-variable in a sample. $\overline{x}$ is the mean of values of the x-variable. ${y}_{i}$ is the value of the y-variable in a sample. $\overline{y}$ is the mean of the values of the y-variable.

But recent research indicates no standard formal classification of the correlation coefficient scales^5^, which suggests using six scales to classify the degree and magnitude of correlation as great (between ± 0.9 and ± 1), very good (between ± 0.75 and ± 0.89), good (between ± 0.5 and ± 0.74), fair (between ± 0.3 and ± 0.49), poor (between ± 0.0 and <  ± 0.29), and no correlation (zero). A correlation value of ± 0.3 or above indicates a correlation between two variables.

### Verification analysis

Verification analysis aims to investigate whether first-round data analysis outcomes might be accepted using second-round datasets simultaneously and whether second-round data analysis results might be accepted using first-round datasets. Repeated reliability and validity analysis would be conducted for the outcomes of the verification analysis between gas and gas, gas and temperature, and gas and wind.

Based on the above verification outcomes, a correlation analysis is then conducted to test and evaluate whether the degree of a strong relationship exists between two variables separately: gas and gas, gas and temperature, and gas and wind. A correlation value of ± 0.3 or above is also used to measure a correlation between two variables.

## Results

The first-round analysis uses obtained data between 00:00:00 am and 23:59:00 on 5 February 2022 in the Case Study mine. The second-round analysis uses collected data between 00:00:00 am and 23:59:00 on 6 February 2022. Before conducting data analysis, three data cleaning procedures are performed during data pre-processing for the first-round analysis and second-round analysis: eliminating extreme values, outliers, and data standardizing. Measurement errors and distortion of hardware devices in the gas monitoring system cause extreme values and outliers. The data obtained from sensors T4 and T5 are eliminated due to too many zero values due to such two sensors not being used for the working-face in the Case Study mine. But they have not been removed from the gas monitoring system. Hence, both T4 and T5 are not included in this research. But they will be investigated in further studies. Data standardization solves the issues of collecting data from the different sensors with various measurements.

Thus, data obtained from six gas sensors (T1, T2, T3, T6, T7, and T8) and two temperature sensors (WD1 and WD2) are used for the following data analyses. This section presents data analyses to support the technical quality of the datasets, including analysis between gas and gas, gas and temperature, and gas and wind.

### Analysis between gas and gas

Two rounds of analysis between gas and gas data are separately conducted using obtained data on 5 and 6 February 2022.

#### First-round analysis between gas and gas

Data from 5 February 2022 are used for the first-round analysis between gas and gas. The reliability and validity are conducted between six items (T1, T2, T3, T6, T7, and T8) (see steps 3.1 and 3.2 in OnlineAppendix 1). All values of Cronbach’s Alpha are considered to have very good reliability (above 0.6) (see Table 4).

**Table 4:** Shown are the reliability and exploratory factor analyses of the first-round analysis conducted between gas and gas.

Group	Affected sensor	Causing sensors	Cronbach's alpha	KMO	Average communality		Anti-image correlations
1	T1	T3	0.760	0.500	0.807	> 0.5	
2	T2	T3, T6	0.700	0.652	0.626	> 0.5	
3	T3	T1	0.760	0.500	0.807	> 0.5	
4	T6	T8	0.647	0.500	0.739	> 0.5	
5	T7	T8	0.869	0.500	0.884	> 0.5	
6	T8	T7	0.869	0.500	0.884	> 0.5	

All Kaiser–Meyer–Olkin (KMO) values are considered greater (greater than 0.5) in the exploratory factor analysis test. Bartlett’s test of Sphericity is 0.000 (*p* < 0.001). All average communality measures are adequate (greater than 0.6). All anti-image Correlation values are more significant than 0.5. Six correlational groups satisfactorily meet the reliability and exploratory factor analyses.

#### Second-round analysis between gas and gas

Data collected on 6 February 2022 are used for the second-round analysis between gas and gas. The reliability and validity tests are conducted between the above six items (see steps 3.1 and 3.2 in Online Appendix 1). All values of Cronbach’s Alpha are considered to have very good reliability (above 0.7) (see Table 5).

**Table 5:** Shown are the reliability and exploratory factor analyses of the second-round analysis conducted between gas and gas.

Group	Affected sensor	Causing sensors	Cronbach’s alpha	KMO	Average communality	Anti-image correlations
1	T1	T6, T7, T8	0.878	0.826	0.733	> 0.5
2	T2	T6, T7, T8	0.896	0.807	0.763	> 0.5
3	T3	T6	0.747	0.500	0.798	> 0.5
4	T6	T2, T7, T8	0.896	0.807	0.763	> 0.5
5	T7	T2, T6, T8	0.896	0.807	0.763	> 0.5
6	T8	T2, T6, T7	0.896	0.807	0.763	> 0.5

All KMO values are considered to have a greater measure (greater than 0.8). Bartlett’s test of Sphericity is 0.000 (*p* < 0.001). All average communality measures are adequate (greater than 0.7). All anti-image correlation values are more significant than 0.5. Six correlational groups satisfactorily meet the reliability and exploratory factor analyses.

#### Verification analysis between gas and gas

The verification analysis is then conducted to compare the results of the first and second-round analyses to confirm data reliability and validity. Due to the verification analysis using the outcomes of the two-round studies rather than those obtained from the sensors, there is no need to procedure the eliminating extreme values, outliers, and data standardization.

**Figure 3:**
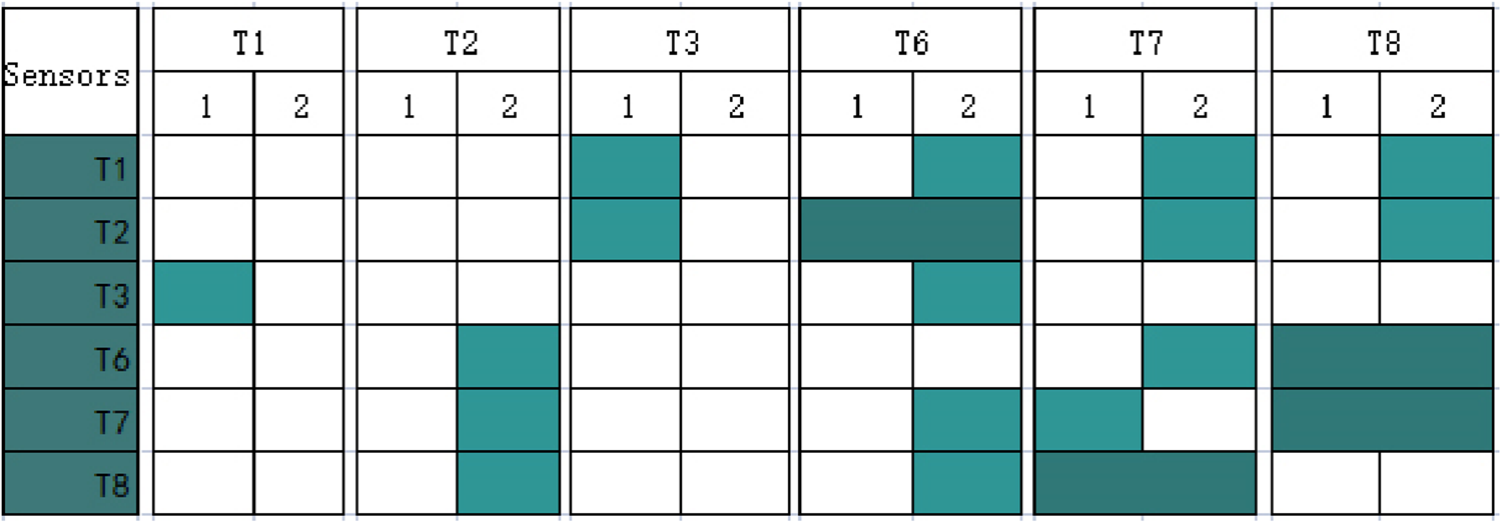
Shown are outcomes between the first- and second-round analyses. The vertical y-axis gives a set of affecting sensors (gas). The horizontal x-axis presents the causing sensors (gas). Light cyan colors the correlational box to indicate existing correlations of T3 and T1 in the first-round analysis and T6 and T2 in the second-round analysis. Deep cyan colors the correlational box to show correlations of T2 and T6, T6 and T8, T7 and T8, and T8 and T7 in both round analyses.

Based on Tables 4 and 5, Fig. 3 compares outcomes between the first- and second-round analyses that indicate four correlational groups, including T2 and T6, T6 and T8, T7 and T8, and T8 and T7.

Repeated reliability and validity are conducted between four correlational groups (see steps 4.1 and 4.2 in Online Appendix 1). All correlational groups satisfactorily meet the reliability and exploratory factor analysis standards. A repeated correlation analysis is followed to test whether correlations exist between items (see Table 6).

**Table 6:** Shown are the outcomes of the correlation analysis conducted between gas and gas. Two good correlations exist between T2 and T6 (0.617) and T6 and T8 (0.653).

	T1	T2	T3	T6	T7	T8
T1						
T2				0.617**		
T3						
T6						0.653**
T7						0.815**
T8					0.815**	

Two very good correlations exist between T7 and T6 (0.815) and T8 and T7 (0.815). This research uses “**” to report *p* values less than 0.001 as *p* < 0.001.

**p<0.01.

**Figure 4:**
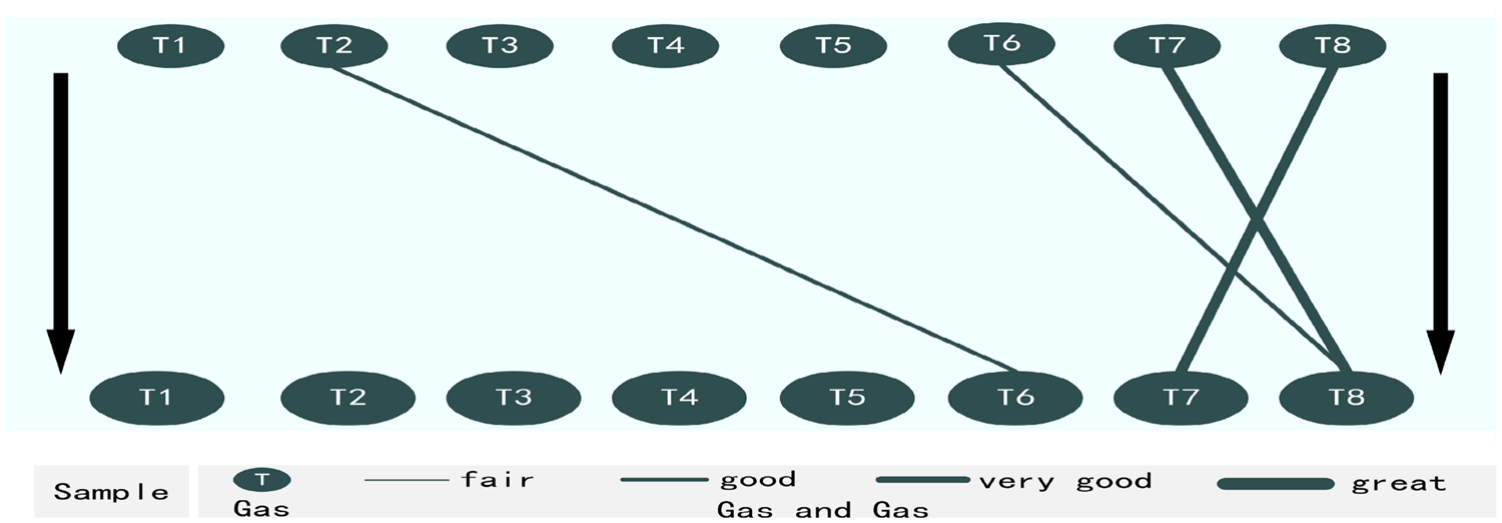
Shown are four significant correlations verified between gas and gas, including two good correlations of T2 and T6 (0.617) and T6 and T8 (0.653), and two very good correlations of T7 and T6 (0.815) and T8 and T7 (0.815).

Thus, the FSV analysis verifies significant correlations exists (T2 and T6, T6 and T8, T7 and T8, and T8 and T7) (see step 5 in Online Appendix 1). The significant correlations between such items are then demonstrated in Fig. 4.

### Analysis between gas and temperature

Two rounds of analysis between gas and temperature data are separately conducted using obtained data on 5 and 6 February 2022.

#### First-round analysis gas and temperature

The first-round analysis is conducted between gas and temperature data collected on 5 February. The results show that six gas items and two temperature items met the standards of the reliability and exploratory factor analyses, including six correlational groups (T1 and WD1, T2 and WD1, T3 and WD1, T6 and WD2, T7 and WD1, and T8 and WD2) (see Table 7). All Cronbach’s Alpha values have very good reliability (above 0.7). Detailed data analyses of six correlational groups are depicted in Tables 8, 9, 10, 11, 12 and 13.

**Table 7:** Shown are the reliability and exploratory factor analyses of the first-round analysis conducted between gas and temperature.

Group	Affected sensors	Causing sensors	Cronbach's alpha	KMO	Average communality	Anti-image correlations
1	T1	WD1	0.880	0.500	0.893	> 0.5
2	T2	WD1	0.936	0.500	0.940	> 0.5
3	T3	WD1	0.886	0.500	0.898	> 0.5
4	T6	WD2	0.868	0.500	0.884	> 0.5
5	T7	WD1	0.734	0.500	0.790	> 0.5
6	T8	WD2	0.802	0.500	0.835	> 0.5

All KMO values are considered a good measure (greater than 0.5). Bartlett’s test of Sphericity is 0.000 (*p* < 0.001). All average communality values are good (greater than 0.7), and Anti-image Correlations are more significant than 0.5.

**Table 8:** Shown are the reliability and exploratory factor analyses conducted between T1 (affected sensor) and WD1 (causing sensor).

Factor		Descriptive statistics					Cronbach's alpha	Validity analysis					
		Mean	Minimum	Maximum	SD	Analysis N		Communalities		Anti-image correlation	Kaiser–Meyer–Olkin measure of sampling adequacy		0.500
								Initial	Extraction				
Affected	T1	0.1343	0.11	0.16	0.01261	1286	0.880	1.000	0.893	0.500^a^	Bartlett's test of sphericity	Approx.Chi-Square	1234.293
												df	1
Causing	WD1	16.877	16.0	17.3	0.3475	1286		1.000	0.893	0.500^a^		Sig	0.000
											Average communalities		0.893

The value of Cronbach's Alpha is 0.88 to have very good reliability (above 0.6). The KMO value shows having a good measure (0.5). Bartlett’s test of Sphericity is 0.000 (*p* < 0.001). The average communality value is 0.893 (greater than 0.5). The value of the Anti-image correlation is also significant (0.5). ^a^Measures of Sampling Adequacy (MSA).

**Table 9:** Shown are the reliability and exploratory factor analyses conducted between T2 (affected sensor) and WD1 (causing sensor).

Factor		Descriptive statistics					Cronbach's alpha	Validity analysis					
		Mean	Minimum	Maximum	SD	Analysis N		Communalities		Anti-image correlation	Kaiser–Meyer–Olkin measure of sampling adequacy		0.500
								Initial	Extraction				
Affected	T2	0.0253	0.02	0.03	0.00500	638	0.936	1.000	0.940	0.500^a^	Bartlett's test of sphericity	Approx.Chi-Square	946.97
												df	1
Causing	WD1	16.000	15.3	16.6	0.5139	638		1.000	0.940	0.500^a^		Sig	0.000
											Average communalities		0.940

The value of Cronbach's Alpha is 0.936 to have great reliability (above 0.6). The KMO value shows having a good measure (0.5). Bartlett’s test of Sphericity is 0.000 (*p* < 0.001). The average communality value is 0.94 (greater than 0.5). The value of the Anti-image correlation is also significant (0.5). ^a^Measures of Sampling Adequacy (MSA).

**Table 10:** Shown are the reliability and exploratory factor analyses conducted between T3 (affected sensor) and WD1 (causing sensor).

Factor		Descriptive statistics					Cronbach's alpha	Validity analysis					
		Mean	Minimum	Maximum	SD	Analysis N		Communalities		Anti-image Correlation	Kaiser–Meyer–Olkin measure of sampling adequacy		0.500
								Initial	Extraction				
Affected	T3	0.0734	0.06	0.09	0.00742	573	0.886	1.000	0.898	0.500^a^	Bartlett's test of sphericity	Approx.Chi-Square	570.835
												df	1
Causing	WD1	16.039	15.3	16.6	0.5226	573		1.000	0.898	0.500^a^		Sig	0.000
											Average communalities		0.898

The value of Cronbach's Alpha is 0.886 to have great reliability (above 0.6). The KMO value shows having a good measure (0.5). Bartlett’s test of Sphericity is 0.000 (*p* < 0.001). The average communality value is 0.898 (greater than 0.5). The value of the Anti-image correlation is also significant (0.5). ^a^Measures of Sampling Adequacy (MSA).

**Table 11:** Shown are the reliability and exploratory factor analyses conducted between T6 (affected sensor) and WD2 (causing sensor).

Factor		Descriptive statistics					Cronbach's alpha	Validity analysis					
		Mean	Minimum	Maximum	SD	Analysis N		Communalities		Anti-image correlation	Kaiser–Meyer–Olkin measure of sampling adequacy		0.500
								Initial	Extraction				
Affected	T6	0.0866	0.04	0.14	0.02089	1508	0.868	1.000	0.884	0.500^a^	Bartlett's test of sphericity	Approx.Chi-Square	1338.856
												df	1
Causing	WD2	16.965	16.8	17.3	0.2352	1508		1.000	0.884	0.500^a^		Sig	0.000
											Average communalities		0.884

The value of Cronbach's Alpha is 0.868 to have very good reliability (above 0.6). The KMO value shows having a good measure (0.5). Bartlett’s test of Sphericity is 0.000 (*p* < 0.001). The average communality value is 0.884 (greater than 0.5). The value of the Anti-image correlation is also significant (0.5). ^a^Measures of Sampling Adequacy (MSA).

**Table 12:** Shown are the reliability and exploratory factor analyses conducted between T2 (affected sensor) and WD1 (causing sensor).

Factor		Descriptive statistics					Cronbach's alpha	Validity analysis					
		Mean	Minimum	Maximum	SD	Analysis N		Communalities		Anti-image correlation	Kaiser–Meyer–Olkin measure of sampling adequacy		0.500
								Initial	Extraction				
Affected	T7	0.1196	0.10	0.18	0.01592	1139	0.734	1.000	0.790	0.500^a^	Bartlett's test of sphericity	Approx.Chi-Square	465.999
												df	1
Causing	WD1	16.848	16.0	17.3	0.3593	1139		1.000	0.790	0.500^a^		Sig	0.000
											Average communalities		0.790

The value of Cronbach's Alpha is 0.734 to have good reliability (above 0.6). The KMO value shows having a good measure (0.5). Bartlett’s test of Sphericity is 0.000 (*p* < 0.001). The average communality value is 0.79 (greater than 0.5). The value of the Anti-image correlation is also significant (0.5). ^a^Measures of Sampling Adequacy (MSA).

**Table 13:** Shown are the reliability and exploratory factor analyses conducted between T8 (affected sensor) and WD2 (causing sensor).

Factor		Descriptive statistics					Cronbach's alpha	Validity analysis					
		Mean	Minimum	Maximum	SD	Analysis N		Communalities		Anti-image correlation	Kaiser–Meyer–Olkin measure of sampling adequacy		0.500
								Initial	Extraction				
Affected	T8	0.0973	0.08	0.11	0.00719	1458	0.835	1.000	0.835	0.500^a^	Bartlett's test of sphericity	Approx.Chi-Square	863.810
												df	1
Causing	WD2	16.953	16.8	17.3	0.2306	1458		1.000	0.835	0.500^a^		Sig	0.000
											Average communalities		0.835

The value of Cronbach's Alpha is 0.835 to have very good reliability (above 0.6). The KMO value shows having a good measure (0.5). Bartlett’s test of Sphericity is 0.000 (*p* < 0.001). The average communality value is 0.835 (greater than 0.5). The value of the Anti-image correlation is also significant (0.5). ^a^Measures of Sampling Adequacy (MSA).

#### Second-round analysis gas and temperature

The second-round analysis of gas and temperature is based on data collected on 6 February. The results show that six gas items and two temperature items meet the standards of the reliability and exploratory factor analyses, including six correlational groups (T1 and WD2, T2 and WD2, T3 and WD1, T6 and WD2, T7 and WD2, and T8 and WD2) (see Table 14). Detailed data analysis of six groups is shown in Tables 15, 16, 17, 18, 19 and 20.

**Table 14:** Shown are the reliability and exploratory factor analyses of the second-round analysis conducted between gas and temperature.

Group	Affected sensors	Causing sensors	Cronbach's alpha	KMO	Average communality	Anti-image correlations
1	T1	WD2	0.832	0.500	0.856	> 0.5
2	T2	WD2	0.748	0.500	0.798	> 0.5
3	T3	WD1	0.638	0.500	0.734	> 0.5
4	T6	WD2	0.670	0.500	0.752	> 0.5
5	T7	WD2	0.749	0.500	0.800	> 0.5
6	T8	WD2	0.739	0.500	0.793	> 0.5

All Cronbach’s Alpha values have very good reliability (above 0.6). All KMO values demonstrate having a greater measure (0.5). Bartlett’s test of Sphericity is 0.000 (*p* < 0.001). All average communality measures are adequate (greater than 0.7). Anti-image Correlations values are more significant than 0.5.

**Table 15:** Shown are the reliability and exploratory factor analyses of the second-round analysis conducted between T1 (affected sensor) and WD2 (causing sensor).

Factor		Descriptive statistics					Cronbach's alpha	Validity analysis					
		Mean	Minimum	Maximum	SD	Analysis N		Communalities		Anti-image correlation	Kaiser–Meyer–Olkin measure of sampling adequacy		0.500
								Initial	Extraction				
Affected	T1	0.1890	0.14	0.36	0.01261	1677	0.832	1.000	0.856	0.500^a^	Bartlett's Test of Sphericity	Approx.Chi-Square	1185.365
												df	1
Causing	WD2	16.988	16.8	17.3	0.2424	1677		1.000	0.856	0.500^a^		Sig	0.000
											Average Communalities		0.856

The value of Cronbach's Alpha is 0.832 to have very good reliability (above 0.6). The KMO value shows having a good measure (0.5). Bartlett’s test of Sphericity is 0.000 (*p* < 0.001). The average communality value is 0.856 (greater than 0.5). The value of the Anti-image correlation is also significant (0.5). ^a^Measures of Sampling Adequacy (MSA).

**Table 16:** Shown are the reliability and exploratory factor analyses of the second-round analysis conducted between T2 (affected sensor) and WD2 (causing sensor).

Factor		Descriptive statistics					Cronbach's alpha	Validity analysis					
		Mean	Minimum	Maximum	SD	Analysis N		Communalities		Anti-image correlation	Kaiser–Meyer–Olkin measure of sampling adequacy		0.500
								Initial	Extraction				
Affected	T2	0.1890	0.02	0.04	0.00477	3622	0.748	1.000	0.856	0.500^a^	Bartlett's test of sphericity	Approx.Chi-Square	1185.365
												df	1
Causing	WD2	16.899	16.8	17.3	0.1996	3622		1.000	0.856	0.500^a^		Sig	0.000
											Average communalities		0.856

The value of Cronbach's Alpha is 0.748 to have good reliability (above 0.6). The KMO value shows having a good measure (0.5). Bartlett’s test of Sphericity is 0.000 (*p* < 0.001). The average communality value is 0.856 (greater than 0.5). The value of the Anti-image correlation is also significant (0.5). ^a^Measures of Sampling Adequacy (MSA).

**Table 17:** Shown are the reliability and exploratory factor analyses of the second-round analysis conducted between T3 (affected sensor) and WD2 (causing sensor).

Factor		Descriptive statistics					Cronbach's alpha	Validity analysis					
		Mean	Minimum	Maximum	SD	Analysis N		Communalities		Anti-image correlation	Kaiser–Meyer–Olkin measure of sampling adequacy		0.500
								Initial	Extraction				
Affected	T3	0.0713	0.05	0.10	0.01198	579	0.638	1.000	0.734	0.500^a^	Bartlett's test of sphericity	Approx.Chi-Square	143.184
												df	1
Causing	WD1	17.351	17.3	17.4	0.0500	579		1.000	0.734	0.500^a^		Sig	0.000
											Average communalities		0.734

The value of Cronbach's Alpha is 0.638 to have good reliability (above 0.6). The KMO value shows having a good measure (0.5). Bartlett’s test of Sphericity is 0.000 (*p* < 0.001). The average communality value is 0.734 (greater than 0.5). The value of the Anti-image correlation is also significant (0.5). ^a^Measures of Sampling Adequacy (MSA).

**Table 18:** Shown are the reliability and exploratory factor analyses of the second-round analysis conducted between T6 (affected sensor) and WD2 (causing sensor).

Factor		Descriptive statistics					Cronbach's alpha	Validity analysis					
		Mean	Minimum	Maximum	SD	Analysis N		Communalities		Anti-image correlation	Kaiser–Meyer–Olkin measure of sampling adequacy		0.500
								Initial	Extraction				
Affected	T6	0.0765	0.04	0.21	0.03237	3490	0.670	1.000	0.752	0.500^a^	Bartlett's test of sphericity	Approx.Chi-Square	1022.100
												df	1
Causing	WD2	16.889	16.8	17.3	0.1911	3490		1.000	0.752	0.500^a^		Sig	0.000
											Average communalities		0.752

The value of Cronbach's Alpha is 0.672 to have good reliability (above 0.6). The KMO value shows having a good measure (0.5). Bartlett’s test of Sphericity is 0.000 (*p* < 0.001). The average communality value is 0.752 (greater than 0.5). The value of the Anti-image correlation is also significant (0.5). ^a^Measures of Sampling Adequacy (MSA).

**Table 19:** Shown are the reliability and exploratory factor analyses of the second-round analysis conducted between T7 (affected sensor) and WD2 (causing sensor).

Factor		Descriptive statistics					Cronbach's alpha	Validity analysis					
		Mean	Minimum	Maximum	SD	Analysis N		Communalities		Anti-image correlation	Kaiser–Meyer–Olkin measure of sampling adequacy		0.500
								Initial	Extraction				
Affected	T7	0.1269	0.10	0.22	0.02201	3417	0.749	1.000	0.800	0.500^a^	Bartlett's test of sphericity	Approx.Chi-Square	1519.439
												df	1
Causing	WD2	16.877	16.8	17.3	0.1806	3417		1.000	0.800	0.500^a^		Sig	0.000
											Average communalities		0.800

The value of Cronbach's Alpha is 0.749 to have good reliability (above 0.6). The KMO value shows having a good measure (0.5). Bartlett’s test of Sphericity is 0.000 (*p* < 0.001). The average communality value is 0.8 (greater than 0.5). The value of the Anti-image correlation is also significant (0.5). ^a^Measures of Sampling Adequacy (MSA).

**Table 20:** Shown are the reliability and exploratory factor analyses of the second-round analysis conducted between T8 (affected sensor) and WD2 (causing sensor).

Factor		Descriptive statistics					Cronbach's alpha	Validity analysis					
		Mean	Minimum	Maximum	SD	Analysis N		Communalities		Anti-image correlation	Kaiser–Meyer–Olkin measure of sampling adequacy		0.500
								Initial	Extraction				
Affected	T8	0.098	0.1	0.2	0.0192	3431	0.739	1.000	0.793	0.500^a^	Bartlett's test of sphericity	Approx.Chi-Square	1439.594
												df	1
Causing	WD2	16.879	16.8	17.3	0.1820	3431		1.000	0.793	0.500^a^		Sig	0.000
											Average communalities		0.793

The value of Cronbach's Alpha is 0.739 to have good reliability (above 0.6). The KMO value shows having a good measure (0.5). Bartlett’s test of Sphericity is 0.000 (*p* < 0.001). The average communality value is 0.793 (greater than 0.5). The value of the Anti-image correlation is also significant (0.5). ^a^Measures of Sampling Adequacy (MSA).

#### Verification analysis gas and temperature

**Figure 5:**
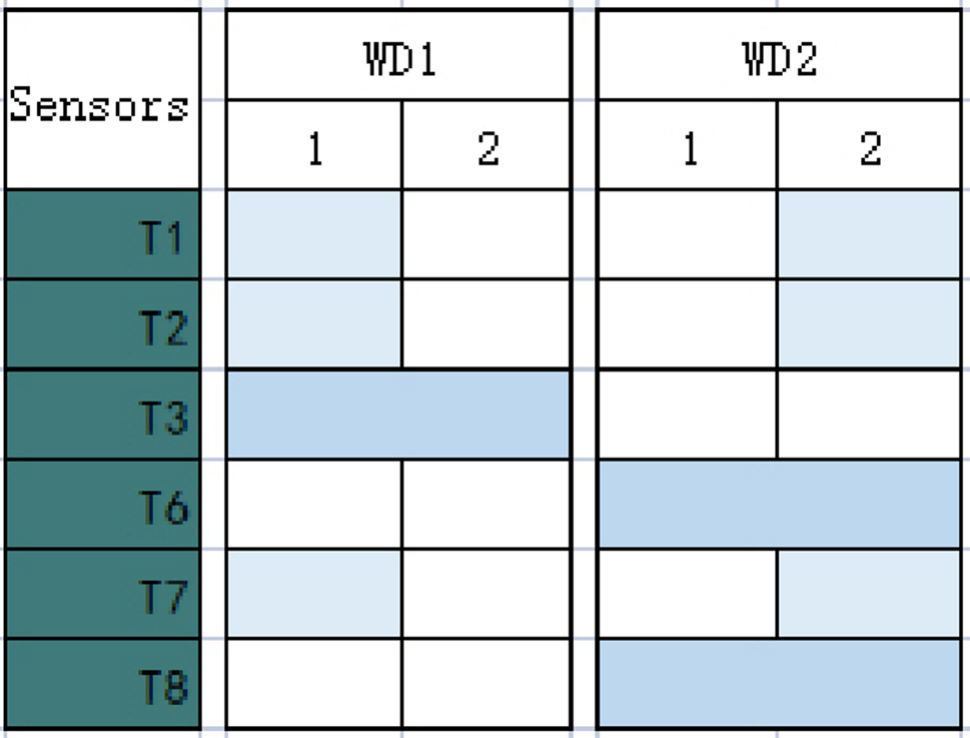
Shown are the outcomes of two-round analyses between gas and temperature. The vertical y-axis gives a set of affecting sensors (gas). The horizontal x-axis presents the causing sensors (temperature). Light blue colors the correlational box to indicate correlations existed of T1 and WD1, T2 and WD1, and T7 and WD1 in the first round, and T1 and WD2, T2 and WD2, and T7 and WD2 in the second round. In both two-round analyses, deep blue colors the correlational box to indicate the correlations between T2 and WD1, T6 and WD2, and T8 and WD2.

Verification analysis is then conducted to compare the results of the first and second-round analyses to confirm data reliability and validity. Based on Tables 7 and 14, Fig. 5 compares the outcomes of two-round analyses between gas and temperature.

Repeated reliability and validity analyses are conducted to confirm that the above three correlational groups (T3 and WD1, T6 and WD2, and T8 and WD2) satisfactorily meet the data analysis standards. A repeated correlation analysis is followed to test whether significant correlations exist between the above groups (T3 and WD1, T6 and WD2, and T8 and WD2) (Table 21).

**Table 21:** Shown are the outcomes of the correlation analysis conducted between gas and temperature.

	WD1	WD2
T3	0.795**	
T6		0.768**
T8		0.669**

Two very good correlations exist between T3 and WD1 (0.795) and T6 and WD2 (0.768). T8 and WD2 have a good correlation (0.669). This research uses “**” to report *p* values less than 0.001 as *p* < 0.001.

**p < 0.01.

**Figure 6:**
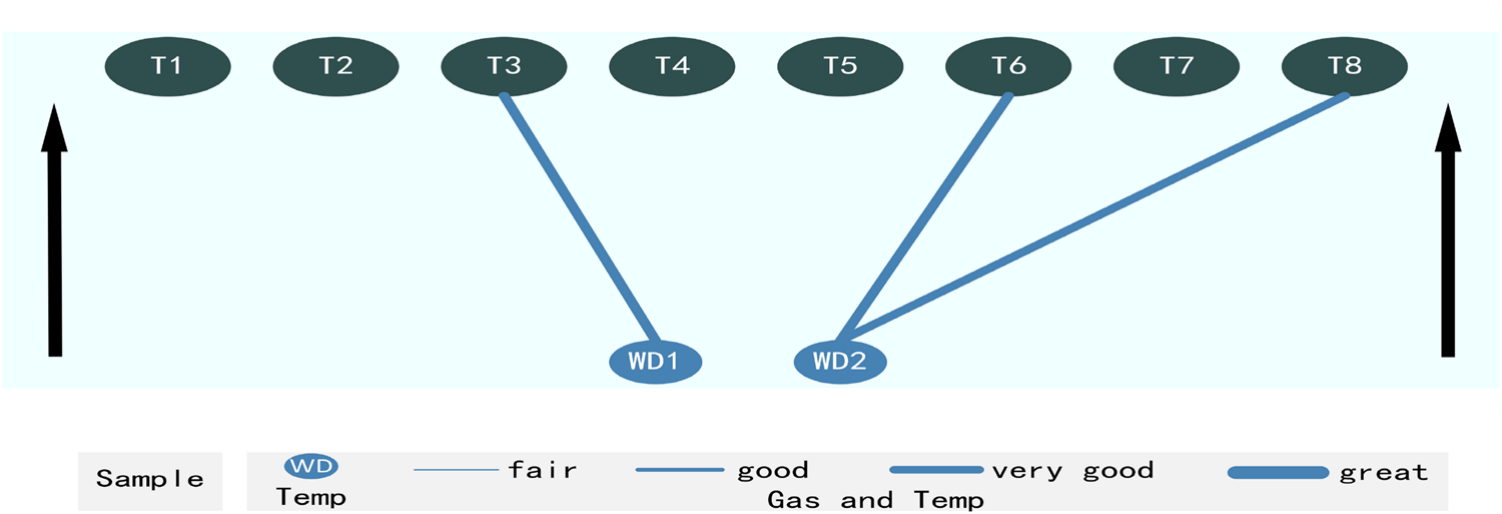
Shown are three significant correlations verified between gas and temperature wind, including a very good correlation of T3 and WD1 (0.795), a very good correlation of T6 and WD2 (0.768), and a good correlation of T8 and WD2 (0.669).

Hence, the FSV analysis verifies significant correlations between T3 and WD1, T6 and WD2, and T8 and WD2 (see Fig. 6).

### Analysis between gas and wind

Two rounds of analysis between gas and wind data are separately conducted using obtained data on 5 and 6 February 2022.

#### First-round analysis between gas and wind

The first-round analysis of gas and wind is based on data collected on 5 February. The results show that six gas items and two wind items meet the standards of the reliability and exploratory factor analyses, including six correlational groups (T1, FS1 and FS2, T2, FS1 and FS2, T3, FS1 and FS2, T6, FS1 and FS2, T7, FS1 and FS2, and T8, FS1, and FS2) (see Table 22). Detailed data analysis of six groups is shown in Tables 23, 24, 25, 26, 27 and 28.

**Table 22:** Shown are the reliability and exploratory factor analyses of the first-round analysis conducted between gas and wind.

Group	Affected sensor	Causing sensor	Cronbach's alpha	KMO	Average communality	Anti-image correlations
1	T1	FS1, FS2	0.676	0.633	0.609	> 0.5
2	T2	FS1, FS2	0.627	0.642	0.573	> 0.5
3	T3	FS1, FS2	0.669	0.639	0.603	> 0.5
4	T6	FS1, FS2	0.729	0.681	0.649	> 0.5
5	T7	FS1, FS2	0.640	0.641	0.582	> 0.5
6	T8	FS1, FS2	0.666	0.638	0.600	> 0.5

All Cronbach’s Alpha values have good reliability (above 0.6). All KMO values demonstrate having a greater measure (greater than 0.5). Bartlett’s test of Sphericity is 0.000 (*p* < 0.001). All average communality measures are adequate (greater than 0.5). Anti-image Correlations values are more significant than 0.5.

**Table 23:** Shown are the reliability and exploratory factor analyses of the first-round analysis conducted between T1 (affected sensor) and causing sensors (FS1 and FS2). The value of Cronbach's Alpha is 0.676 to have good reliability (above 0.6).

Factor		Descriptive statistics					Cronbach's alpha	Validity analysis					
		Mean	Minimum	Maximum	SD	Analysis N		Communalities		Anti-image correlation	Kaiser–Meyer–Olkin measure of sampling adequacy		0.633
								Initial	Extraction				
Affected	T1	0.1449	0.13	0.16	0.00662	598	0.676	1.000	0.623	0.598^a^	Bartlett's test of sphericity	Approx.Chi-Square	296.487
Causing	FS1	0.9327	0.89	1.00	0.02351	598		1.000	0.548	0.695^a^		df	3
	FS2	0.6285	0.37	0.85	0.09000	598		1.000	0.549	0.634^a^		Sig	0.000
											Average communalities		0.609

The KMO value shows having a good measure (0.633). Bartlett’s test of Sphericity is 0.000 (*p* < 0.001). The average communality value is 0.609 (greater than 0.5). All Anti-image correlation values are also significant (great than 0.5). ^a^Measures of Sampling Adequacy (MSA).

**Table 24:** Shown are the reliability and exploratory factor analyses of the first-round analysis conducted between T2 (affected sensor) and two causing sensors (FS1 and FS2).

Factor		Descriptive statistics					Cronbach's alpha	Validity analysis					
		Mean	Minimum	Maximum	SD	Analysis N		Communalities		Anti-image correlation	Kaiser–Meyer–Olkin measure of sampling adequacy		0.642
								Initial	Extraction				
Affected	T2	0.0324	0.02	0.04	0.00473	598	0.676	1.000	0.623	0.619^a^	Bartlett's test of sphericity	Approx.Chi-Square	211.949
Causing	FS1	0.9327	0.89	1.00	0.02351	598		1.000	0.548	0.657^a^		df	3
	FS2	0.6285	0.37	0.85	0.09000	598		1.000	0.549	0.656^a^		Sig	0.000
											Average communalities		0.573

The value of Cronbach's Alpha is 0.676 to have good reliability (above 0.6). The KMO value shows having a good measure (0.642). Bartlett’s test of Sphericity is 0.000 (*p* < 0.001). The average communality value is 0.573 (greater than 0.5). All Anti-image correlation values are also significant (great than 0.5). ^a^Measures of Sampling Adequacy (MSA).

**Table 25:** Shown are the reliability and exploratory factor analyses of the first-round analysis conducted between T3 (affected sensor) and two causing sensors (FS1 and FS2).

Factor		Descriptive statistics					Cronbach's alpha	Validity analysis					
		Mean	Minimum	Maximum	SD	Analysis N		Communalities		Anti-image correlation	Kaiser–Meyer–Olkin measure of sampling adequacy		0.639
								Initial	Extraction				
Affected	T3	0.0733	0.06	0.09	0.00736	593	0.669	1.000	0.685	0.606^a^	Bartlett's test of sphericity	Approx.Chi-Square	276.211
Causing	FS1	0.9328	0.89	1.00	0.02358	593		1.000	0.539	0.682^a^		df	3
	FS2	0.6280	0.37	0.85	0.08981	593		1.000	0.585	0.649^a^		Sig	0.000
											Average communalities		0.603

The value of Cronbach's Alpha is 0.669 to have good reliability (above 0.6). The KMO value shows having a good measure (0.639). Bartlett’s test of Sphericity is 0.000 (*p* < 0.001). The average communality value is 0.603 (greater than 0.5). All Anti-image correlation values are also significant (great than 0.5). ^a^Measures of Sampling Adequacy (MSA).

**Table 26:** Shown are the reliability and exploratory factor analyses of the first-round analysis conducted between T6 (affected sensor) and two causing sensors (FS1 and FS2).

Factor		Descriptive statistics					Cronbach's alpha	Validity analysis					
		Mean	Minimum	Maximum	SD	Analysis N		Communalities		Anti-image correlation	Kaiser–Meyer–Olkin measure of sampling adequacy		0.681
								Initial	Extraction				
Affected	T6	0.0719	0.06	0.08	0.00559	200	0.729	1.000	0.674	0.664^a^	Bartlett's test of sphericity	Approx.Chi-Square	122.188
Causing	FS1	0.9303	0.90	1.00	0.02342	200		1.000	0.618	0.706^a^		df	3
	FS2	0.6278	0.36	0.84	0.09858	200		1.000	0.654	0.678^a^		Sig	0.000
											Average communalities		0.649

The value of Cronbach's Alpha is 0.669 to have good reliability (above 0.6). The KMO value shows having a good measure (0.639). Bartlett’s test of Sphericity is 0.000 (*p* < 0.001). The average communality value is 0.603 (greater than 0.5). All Anti-image correlation values are also significant (great than 0.5). ^a^Measures of Sampling Adequacy (MSA).

**Table 27:** Shown are the reliability and exploratory factor analyses of the first-round analysis conducted between T7 (affected sensor) and two causing sensors (FS1 and FS2).

Factor		Descriptive statistics					Cronbach's alpha	Validity analysis					
		Mean	Minimum	Maximum	SD	Analysis N		Communalities		Anti-image correlation	Kaiser–Meyer–Olkin measure of sampling adequacy		0.641
								Initial	Extraction				
Affected	T7	0.1482	0.11	0.24	0.03630	598	0.640	1.000	0.643	0.614^a^	Bartlett's test of sphericity	Approx.Chi-Square	229.912
Causing	FS1	0.9327	0.89	1.00	0.02351	598		1.000	0.536	0.671^a^		df	3
	FS2	0.6285	0.37	0.85	0.09000	598		1.000	0.567	0.650^a^		Sig	0.000
											Average communalities		0.582

The value of Cronbach's Alpha is 0.640 to have good reliability (above 0.6). The KMO value shows having a good measure (0.641). Bartlett’s test of Sphericity is 0.000 (*p* < 0.001). The average communality value is 0.582 (greater than 0.5). All Anti-image correlation values are also significant (great than 0.5). ^a^Measures of Sampling Adequacy (MSA).

**Table 28:** Shown are the reliability and exploratory factor analyses of the first-round analysis conducted between T8 (affected sensor) and two causing sensors (FS1 and FS2).

Factor		Descriptive statistics					Cronbach's alpha	Validity analysis					
		Mean	Minimum	Maximum	SD	Analysis N		Communalities		Anti-image correlation	Kaiser–Meyer–Olkin measure of sampling adequacy		0.638
								Initial	Extraction				
Affected	T8	0.1246	0.09	0.20	0.02736	598	0.666	1.000	0.683	0.604^a^	Bartlett's test of sphericity	Approx.Chi-Square	272.384
Causing	FS1	0.9327	0.89	1.00	0.02351	598		1.000	0.543	0.675^a^		df	3
	FS2	0.6285	0.37	0.85	0.09000	598		1.000	0.575	0.653^a^		Sig	0.000
											Average communalities		0.600

The value of Cronbach's Alpha is 0.666 to have good reliability (above 0.6). The KMO value shows having a good measure (0.638). Bartlett’s test of Sphericity is 0.000 (*p* < 0.001). The average communality value is 0.6 (greater than 0.5). All Anti-image correlation values are also significant (great than 0.5). ^a^Measures of Sampling Adequacy (MSA).

#### Second-round analysis between gas and wind

The second-round gas and wind data analysis is based on data collected on 6 February. The results show that six gas items and three wind items meet the standards of the reliability and exploratory factor analyses, including six correlational groups (T1 and FS3, T2 and FS1, T3 and FS2, T6 and FS1, T7 and FS1, and T8 and FS1) (see Table 29). All Cronbach’s Alpha values have very good reliability (above 0.6). All KMO values demonstrate a greater measure (greater than 0.5). Bartlett’s test of Sphericity is 0.000 (*p* < 0.001). All average communality measures are adequate (greater than 0.7). Anti-image Correlations values are significant (more than 0.5). Detailed data analysis of six groups is shown in Tables 30, 31, 32, 33, 34 and 35.

**Table 29:** Shown are the reliability and exploratory factor analyses of the second-round analysis conducted between gas and wind.

Group	Affected Sensor	Causing Sensor	Cronbach's Alpha	KMO	Average Communality	Anti-image Correlations
1	T1	FS3	0.626	0.500	0.728	> 0.5
2	T2	FS1	0.800	0.500	0.833	> 0.5
3	T3	FS2	0.605	0.500	0.717	> 0.5
4	T6	FS1	0.670	0.500	0.752	> 0.5
5	T7	FS1	0.902	0.500	0.910	> 0.5
6	T8	FS1	0.831	0.500	0.856	> 0.5

All Cronbach’s Alpha values have very good reliability (above 0.6). All KMO values demonstrate having a greater measure (0.5). Bartlett’s test of Sphericity is 0.000 (*p* < 0.001). All average communality measures are adequate (greater than 0.7). Anti-image Correlations values are more significant than 0.5.

**Table 30:** Shown are the reliability and exploratory factor analyses of the second-round analysis conducted between T1 (affected sensor) and FS3 (causing sensor).

Factor		Descriptive statistics					Cronbach's alpha	Validity analysis					
		Mean	Minimum	Maximum	SD	Analysis N		Communalities		Anti-image correlation	Kaiser–Meyer–Olkin measure of sampling adequacy		0.500
								Initial	Extraction				
Affected causing	T1	0.1528	0.14	0.17	0.00815	197	0.626	1.000	0.728	0.500^a^	Bartlett's test of sphericity	Approx.Chi-Square	45.150
												df	1
Causing	FS3	0.5379	0.51	0.61	0.02733	197		1.000	0.728	0.500^a^		Sig	0.000
											Average communalities		0.728

The value of Cronbach's Alpha is 0.626 to have good reliability (above 0.6). The KMO value shows having a good measure (0.5). Bartlett’s test of Sphericity is 0.000 (*p* < 0.001). The average communality value is 0.835 (greater than 0.5). The value of Anti-image correlation value is also significant (0.5). ^a^Measures of Sampling Adequacy (MSA).

**Table 31:** Shown are the reliability and exploratory factor analyses of the second-round analysis conducted between T2 (affected sensor) and FS1 (causing sensor).

Factor		Descriptive statistics					Cronbach's alpha	Validity analysis					
		Mean	Minimum	Maximum	SD	Analysis N		Communalities		Anti-image correlation	Kaiser–Meyer–Olkin measure of sampling adequacy		0.500
								Initial	Extraction				
Affected causing	T2	0.1528	0.14	0.17	0.00815	197	0.800	1.000	0.833	0.500^a^	Bartlett's test of sphericity	Approx.Chi-Square	98.582
												df	1
Causing	FS1	0.9140	0.91	0.92	0.00491	197		1.000	0.833	0.500^a^		Sig	0.000
											Average communalities		0.833

The value of Cronbach's Alpha is 0.8 to have good reliability (above 0.6). The KMO value shows having a good measure (0.5). Bartlett’s test of Sphericity is 0.000 (*p* < 0.001). The average communality value is 0.833 (greater than 0.5). The Anti-image correlation value is also significant (0.5). ^a^Measures of Sampling Adequacy (MSA).

**Table 32:** Shown are the reliability and exploratory factor analyses of the second-round analysis conducted between T3 (affected sensor) and FS2 (causing sensor).

Factor		Descriptive statistics					Cronbach's alpha	Validity analysis					
		Mean	Minimum	Maximum	SD	Analysis N		Communalities		Anti-image correlation	Kaiser–Meyer–Olkin measure of sampling adequacy		0.500
								Initial	Extraction				
Affected causing	T3	0.0671	0.05	0.10	0.00957	432	0.605	1.000	0.717	0.500^a^	Bartlett's test of sphericity	Approx.Chi-Square	89.415
												df	1
Causing	FS2	0.8604	0.51	1.16	0.13340	432		1.000	0.717	0.500^a^		Sig	0.000
											Average communalities		0.717

The value of Cronbach's Alpha is 0.605 to have good reliability (above 0.6). The KMO value shows having a good measure (0.5). Bartlett’s test of Sphericity is 0.000 (*p* < 0.001). The average communality value is 0.717 (greater than 0.5). The Anti-image correlation value is also significant (0.5). ^a^Measures of Sampling Adequacy (MSA).

**Table 33:** Shown are the reliability and exploratory factor analyses of the second-round analysis conducted between T6 (affected sensor) and FS1 (causing sensor).

Factor		Descriptive statistics					Cronbach's alpha	Validity analysis					
		Mean	Minimum	Maximum	SD	Analysis N		Communalities		Anti-image correlation	Kaiser–Meyer–Olkin measure of sampling adequacy		0.500
								Initial	Extraction				
Affected causing	T6	0.122	0.1	0.3	0.0472	432	0.670	1.000	0.752	0.500^a^	Bartlett's test of sphericity	Approx.Chi-Square	125.617
												df	1
Causing	FS1	0.9168	0.91	0.93	0.00859	432		1.000	0.752	0.500^a^		Sig	0.000
											Average communalities		0.752

The value of Cronbach's Alpha is 0.67 to have good reliability (above 0.6). The KMO value shows having a good measure (0.5). Bartlett’s test of Sphericity is 0.000 (*p* < 0.001). The average communality value is 0.752 (greater than 0.5). The Anti-image correlation value is also significant (0.5). ^a^Measures of Sampling Adequacy (MSA).

**Table 34:** Shown are the reliability and exploratory factor analyses of the second-round analysis conducted between T7 (affected sensor) and FS1 (causing sensor).

Factor		Descriptive statistics					Cronbach's alpha	Validity analysis					
		Mean	Minimum	Maximum	SD	Analysis N		Communalities		Anti-image correlation	Kaiser–Meyer–Olkin measure of sampling adequacy		0.500
								Initial	Extraction				
Affected causing	T7	0.1959	0.12	0.38	0.06960	473	0.902	1.000	0.910	0.500^a^	Bartlett's test of sphericity	Approx.Chi-Square	527.281
												df	1
Causing	FS1	0.9171	0.91	0.93	0.00826	473		1.000	0.910	0.500^a^		Sig	0.000
											Average communalities		0.910

The value of Cronbach's Alpha is 0.902 to have great reliability (above 0.6). The KMO value shows having a good measure (0.5). Bartlett’s test of Sphericity is 0.000 (*p* < 0.001). The average communality value is 0.91 (greater than 0.5). The Anti-image correlation value is also significant (0.5). ^a^Measures of Sampling Adequacy (MSA).

**Table 35:** Shown are the reliability and exploratory factor analyses of the second-round analysis conducted between T8 (affected sensor) and FS1 (causing sensor).

Factor		Descriptive statistics					Cronbach's alpha	Validity analysis					
		Mean	Minimum	Maximum	SD	Analysis N		Communalities		Anti-image correlation	Kaiser–Meyer–Olkin measure of sampling adequacy		0.500
								Initial	Extraction				
Affected causing	T8	0.1372	0.10	0.21	0.03102	473	0.831	1.000	0.856	0.500^a^	Bartlett's test of sphericity	Approx.Chi-Square	331.429
												df	1
Causing	FS1	0.9171	0.91	0.93	0.00826	473		1.000	0.856	0.500^a^		Sig	0.000
											Average communalities		0.856

The value of Cronbach's Alpha is 0.831 to have good reliability (above 0.6). The KMO value shows having a good measure (0.5). Bartlett’s test of Sphericity is 0.000 (*p* < 0.001). The average communality value is 0.856 (greater than 0.5). The Anti-image correlation value is also significant (0.5). ^a^Measures of Sampling Adequacy (MSA).

#### Verification analysis between gas and wind

**Figure 7:**
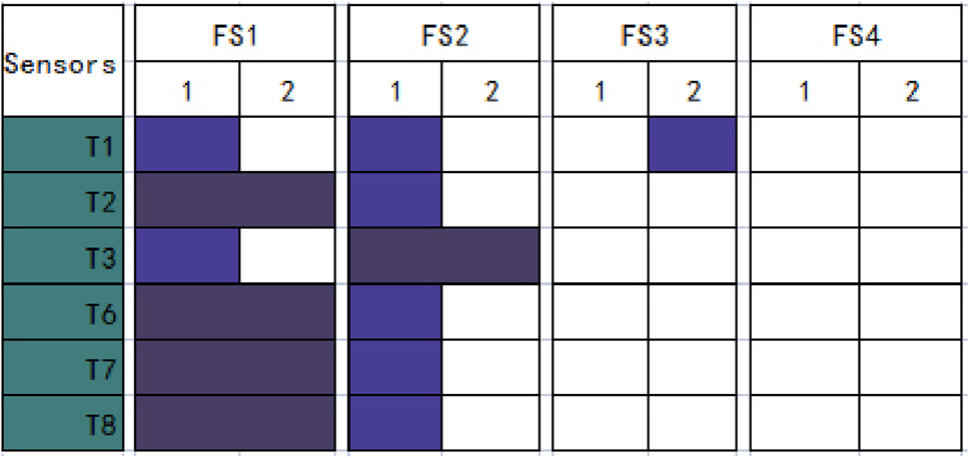
Shown are the outcomes of two-round analyses between gas and wind. The vertical y-axis gives a set of affecting sensors (gas). The horizontal x-axis presents the causing sensors (wind). Light navy colors the correlational box to indicate correlations between T1 and FS1, T1 and FS2, T3 and FS1, T6 and FS2, T7 and FS2, and T8 and FS2) in the first round and T1 and FS3 in the second round. Deep navy colors the correlational box to indicate correlations betweenT2 and FS1, T3 and FS2, T6 and FS1, T7 and FS1, and T8 and TS1 in two-round analyses.

Verification analysis is then conducted to compare the results of the first and second-round analyses to confirm data reliability and validity. Based on Tables 22 and 29, Fig. 7 compares the outcomes of two-round analyses between gas and wind.

Repeated reliability and validity are conducted between five correlational groups. Two groups (T2 and FS1, T7 and FS1) do not meet the data analysis standards. Three correlational groups satisfactorily meet the reliability and exploratory factor analysis standards. They are T3 and FS2, T6 and FS1, and T8 and FS1. A repeated correlation analysis tests whether correlations exist between such items (see Table 36).

**Table 36:** Shown are the outcomes of the correlation analysis conducted between gas and wind.

	FS1	FS2	FS3	FS4
T3		0.467**		
T6	0.468**			
T8	0.428**			

The results indicate three fair correlations—T3 and FS2 (0.467), T6 and FS1 (0.468), and T8 and FS1 (0.428). This research uses “**” to report *p* values less than 0.001 as *p* < 0.001.

**p < 0.01.

**Figure 8:**
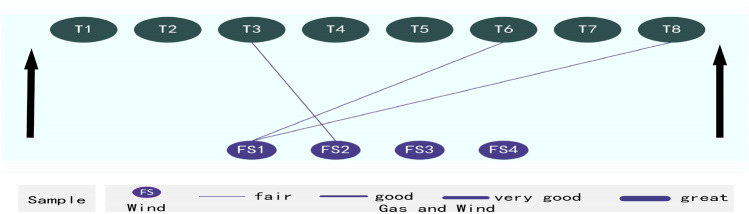
Shown are three fair correlations verified between gas and wind, including T3 and FS2 (0.467), T6 and FS1 (0.468), and T8 and FS1 (0.428).

Thus, the FSV analysis verifies significant correlations (T3 and FS2, T6 and FS1, and T8 and FS1) (see Fig. 8).

## Discussion

**Figure 9:**
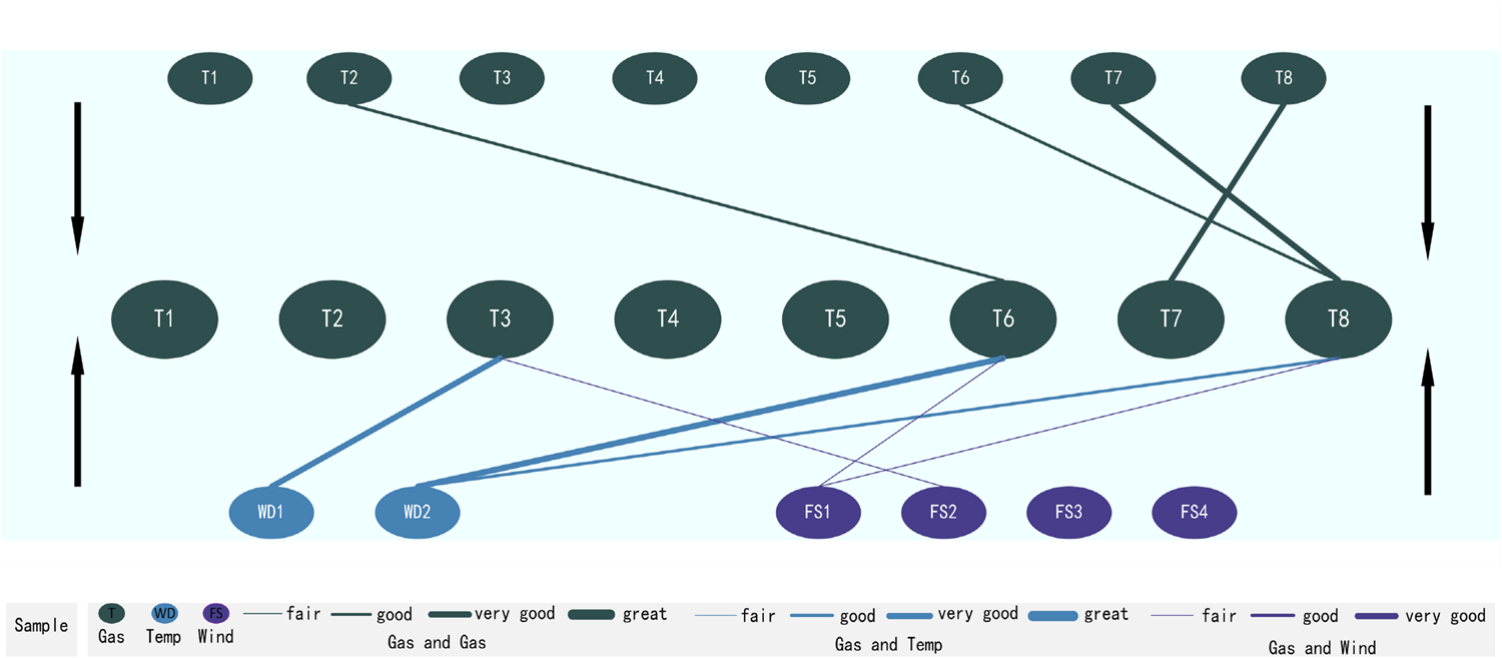
Shown is the Triple-Correlation Analysis model, including ten verified correlations. Four significant correlations exist between gas and gas, including two good correlations between T2 and T6 (0.617) and T6 and T8 (0.653), and two very good correlations between T7 and T6 (0.815) and T8 and T7 (0.815) (see Table 6). Three significant correlations exist between gas and temperature, including two very good correlations between T3 and WD1 (0.795) and T6 and WD2 (0.768), and one good correlation between T8 and WD2 (0.669) (see Table 21). Three fair correlations exist between gas and wind, including T3 and FS2 (0.467), T6 and FS1 (0.468), and T8 and FS1 (0.428) (see Table 36).

Based on Figs. 4, 6, and 8, a triple-correlation analysis model is established for developing a gas warning system in the Case Study mine (see Fig. 9). It incorporates ten verified correlations, including gas and gas (4), gas and temperature (3), and gas and wind (3). The result proves the correlational analysis existed between gas and gas, gas and temperature, and gas and wind.

For enhancing to validate the research outcomes, four additional experiments are also conducted to test whether such correlations existed in different working-faces (no.1217 and no.3209) and other seasons (Summer and Winter) in the Case Study mine (see Table 37 and Dataset 3). All results indicate strong existing correlations between gas and gas, gas and temperature, and gas and wind.

**Table 37:** Shown are four additional experiments conducted to verify the robustness of the Triple-Correlation Analysis Theoretical Framework.

Round	Working-face	Date	Time	Season
1	1217	4-Dec-21	00:00–23:59:59	Winter
2	1217	5-Dec-21	00:00–23:59:59	Winter
3	1217	15-Jun-22	00:00–23:59:59	Summer
4	3209	15-Jun-22	00:00–23:59:59	Summer

The first test was conducted in working-face no.1217 in Case Study mine on 4 Dec 2021 in Winter. The second test was repeated in the same working-face on 5 Dec 2021. The third test was repeated in the working-face no.1217 on 15 Jun 2022 in Summer. The fourth test was conducted on the same day. But it was in the different working-face no.3209.

**Figure 10:**
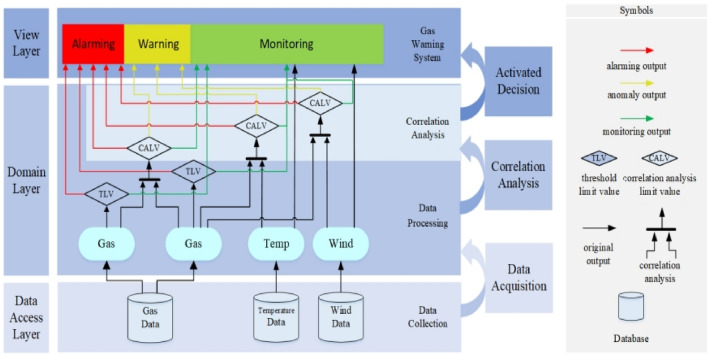
Shown is a UML model of a gas warning system comprised of three layers from the bottom to the top—data access layer, domain layer, and view layer: (1) Data acquisition: This logic flow is run between the Data Access Layer and Domain Layer. The data are obtained from gas, temperature, and wind databases. (2) Correlation analysis: Within the Domain Layer, correlation analyses are conducted separately between data upstream of gas and gas, gas and temperature, and gas and wind. (3) Activated decision: This step bridges the Domain Layer and the View Layer.

Thus, this research uses an explored FSV analysis approach to strongly verify the robustness of the Triple-Correlation Analysis Theoretical Framework for developing a gas warning system to improve the warning systems’ sensitivity and reduce the incidence of gas explosions. To help researchers and practicians understand better the system’s architectural design, a unified modeling language (UML) is developed to demonstrate how this framework is integrated into a gas system^5^, which comprises three layers (data access layer, domain layer, and view layer) and three decision-making rules (see Fig. 10).

Three decision-making rules consist of:

- If the data outputs exceed the TLV, the alarming system would immediately alert the safety-response management team.
- The warning system will inform the safety-responsive team if the real-time correlation analysis value (CAV) exceeds the correlation analysis limit value (CALV) between gas and gas, gas and temperature, or gas and wind. This status does not state any risks, but the safety-response management team must immediately check the monitoring system to identify potential hazards.
- The original data will be forwarded to the monitoring system if the CAV does not exceed the CALV.

**Figure 11:**
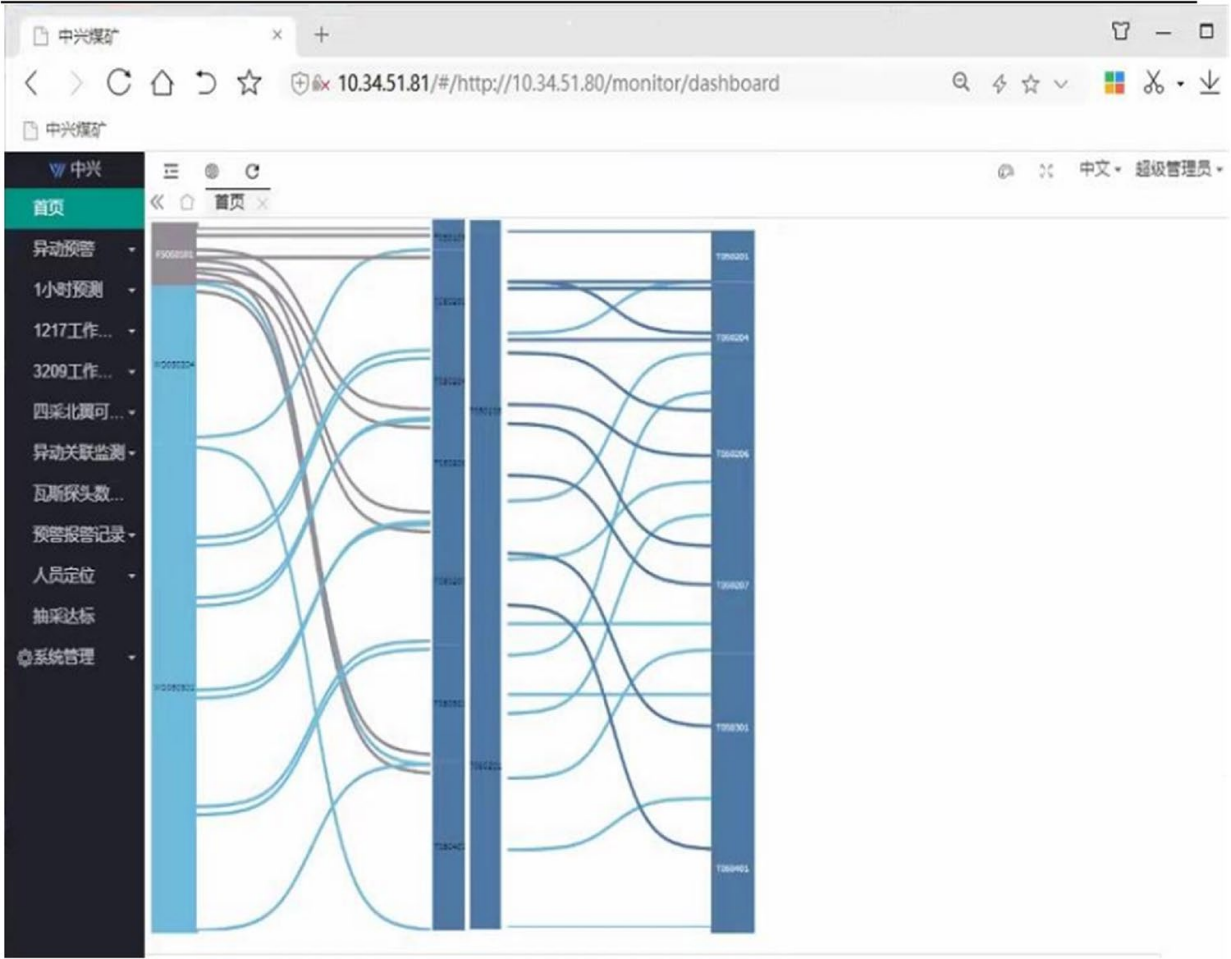
Shown is the system screenshot of the Innovative Integrated Gas Warning System deployed in the Case Study mine. The system integrates the Triple-Correlation Analysis model into the gas monitoring system with incorporated analysis of gas and gas, gas and temperature, and gas and wind. Cyan lines indicate existing correlations between gas and gas. Blue lines indicate existing correlations between gas and temperature. Navy lines indicate existing correlations between gas and wind.

As a result, the Triple-Correlation Analysis model (see Fig. 9) is integrated into the gas monitoring system in the Case Study mine with incorporated analysis of gas and gas, gas and temperature, and gas and wind, which is successfully adopted for developing an Innovative Integrated Gas Warning System in Dec 2021. The system's screenshot is provided in Fig. 11.

## Conclusion

This research aims to explore a proposed FSV analysis approach to verify the robustness of the Trip-Correlation Analysis Theoretical Framework for developing a gas warning system to improve the warning systems’ sensitivity and reduce the incidence of gas explosions. A mixed qualitative and quantitative research methodology is adopted, including a case study and correlational research.

The first-round analysis uses data obtained on 5 February 2022 in the Case Study mine. The second-round analysis uses data collected on 6 February 2022. Verification analysis is then followed to compare the results of the first and second-round analyses to confirm data reliability and validity. Four additional experiments are also conducted to test whether such correlations existed in different working faces (no.1217 and no.3209) and other seasons (Summer and Winter). All tests indicate three significant correlations between gas, temperature, and wind that verify the robustness of the Triple-Correlation Analysis Theoretical Framework (see Fig. 1).

To help researchers and practicians better understand the system’s architectural design, a UML is developed to demonstrate how this framework is integrated into a gas system (see Fig. 10). Pseudocode is also provided to describe the system's implementation, including the system's data analysis and processing logic, which may help researchers in other domains implement the methodology presented in this work (see Online Appendix 7).

The outcomes imply that this framework is potentially valuable for developing other warning systems. The proposed FSV approach can also be adopted for exploring data patterns insightfully to offer new perspectives to develop warning systems for different industry applications. Another finding is that T4 and T5 sensors are not in use due to not being removed from the gas monitoring system. The implication is that they may add to the Trip-Correlation Analysis Theoretical Framework in further research to develop a more sensitive warning system. Using such findings to explore an extended Trip-Correlation Analysis Theoretical Framework in further research is more valuable.

The limitation is that gas, temperature, and wind sensors are regularly changed monthly due to the ongoing mining processing in the Case Study mine. The changes might include hardware relocation, sensor removal, and added detectors. The correlation analysis of data collected from gas, temperature and wind must be re-conducted for any sensor changes following the procedure of the FSV analysis approach. The second limitation is that this research focuses on verifying the robustness of the Trip-Correlation Analysis Theoretical Framework, which incorporates the analysis of gas and gas, gas and temperature, and gas and wind. The ambient conditions remain the same on 5 Feb and 6 Feb 2022. Further research is needed to explore whether other ambient conditions impact the performance and effectiveness of gas warning systems, such as humidity, wind, sunny, cloudy, and even human disturbance. Another limitation is that this research does not consider other data quality issues such as errors in measurement, noise, missing values, etc. They should be solved by updated hardware devices and system algorithms. For example, many studies have provided methods for solving measurement errors^21^. More effective sensors with efficient system algorithms applied to the Trip-Correlation Analysis Theoretical Framework might be used for developing an innovative gas warning system to improve the warning systems’ sensitivity and reduce the incidence of gas explosions. It is valuable to investigate them further.

## Supplementary Information

Supplementary Information 1.

Supplementary Information 2.

Supplementary Information 3.

Supplementary Information 4.

Supplementary Information 5.

Supplementary Information 6.

Supplementary Information 7.

## Data Availability

IBM^®^ SPSS^®^ Statistics version 26 is used for this research to analyse data. This published article and its supplementary information files include all data generated or analyzed during this study. The data supporting the study's findings are available in the public domain Zenodo with license CC BY4.0 from https://zenodo.org/record/6450546, https://zenodo.org/record/6450554, and https://doi.org/10.5281/zenodo.7603551.
