# Supplementary material for: An FSV analysis approach to verify the robustness of the triple-correlation analysis theoretical framework
Source: Sci Rep. 2023 Jun 14;13:9621. doi: 10.1038/s41598-023-35900-3 (PMC10267157; doi:10.1038/s41598-023-35900-3)
Supplement: Supplementary file 1 — Supplementary Information 1. [file 41598_2023_35900_MOESM1_ESM.docx]

**Appendix 1: Overview of FSV Data Analysis Flowchart**

**
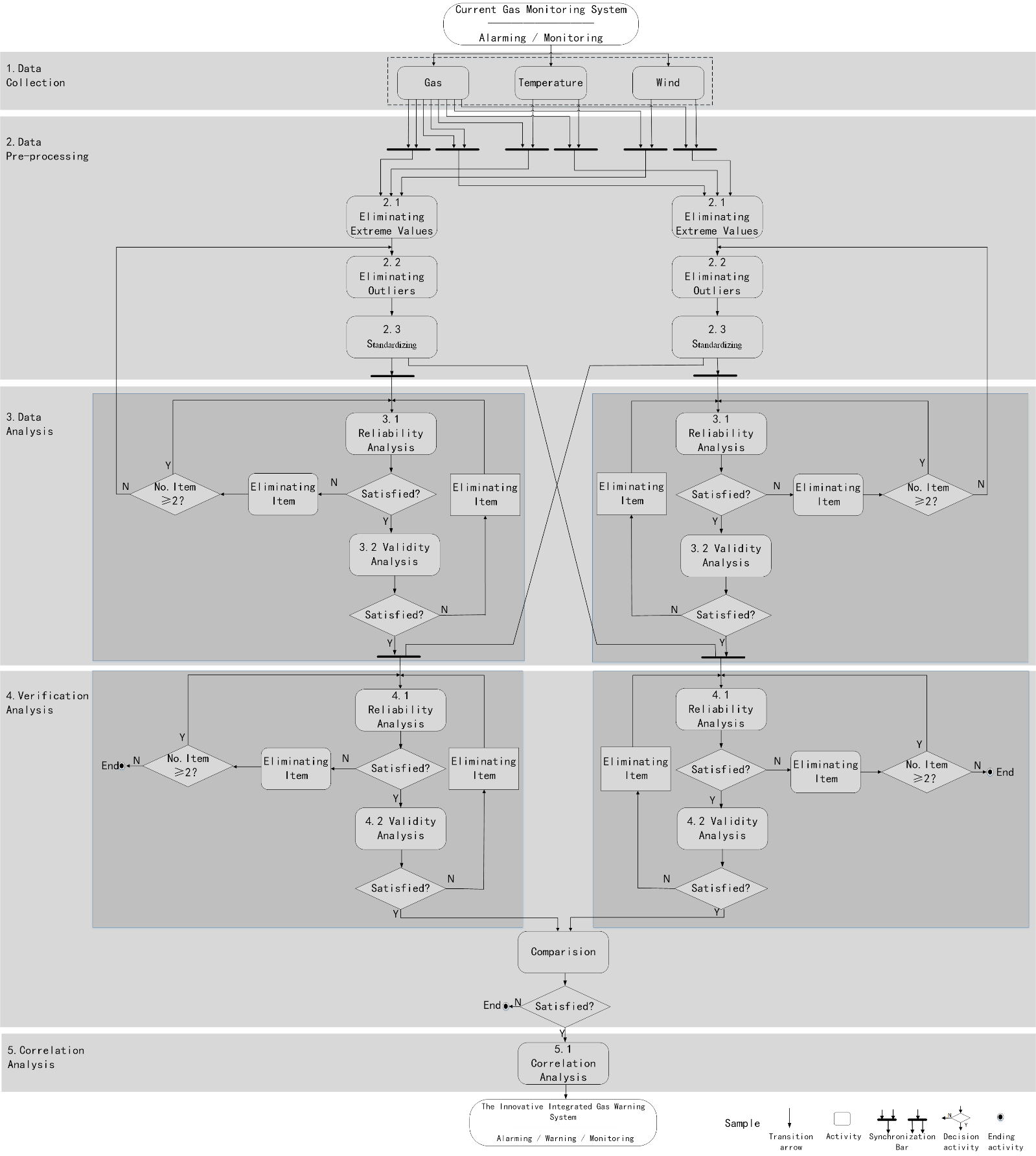
**

**Appendix 2: Gas Data on 5 Feb 2022**


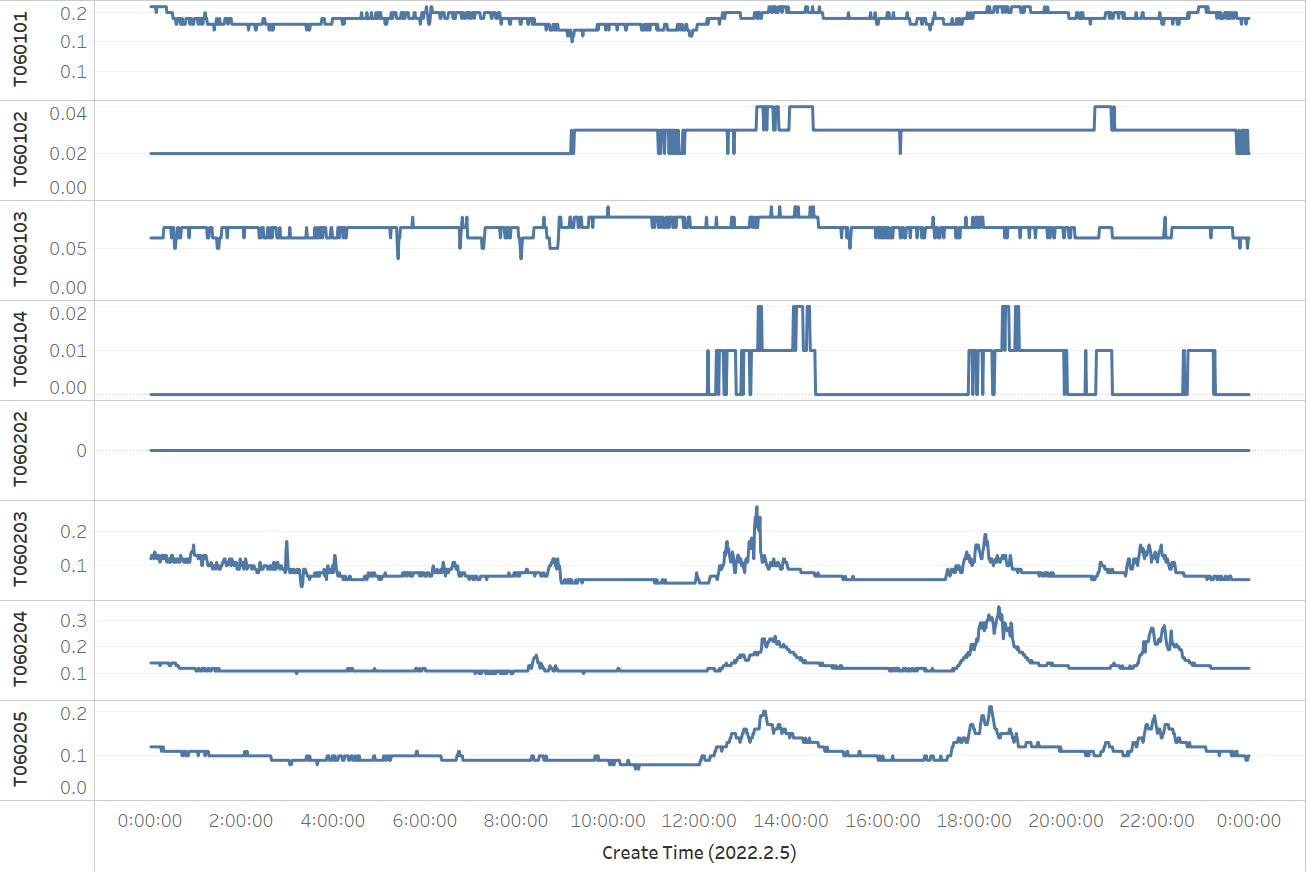


**Appendix 3: Temperature Data on 5 Feb 2022**


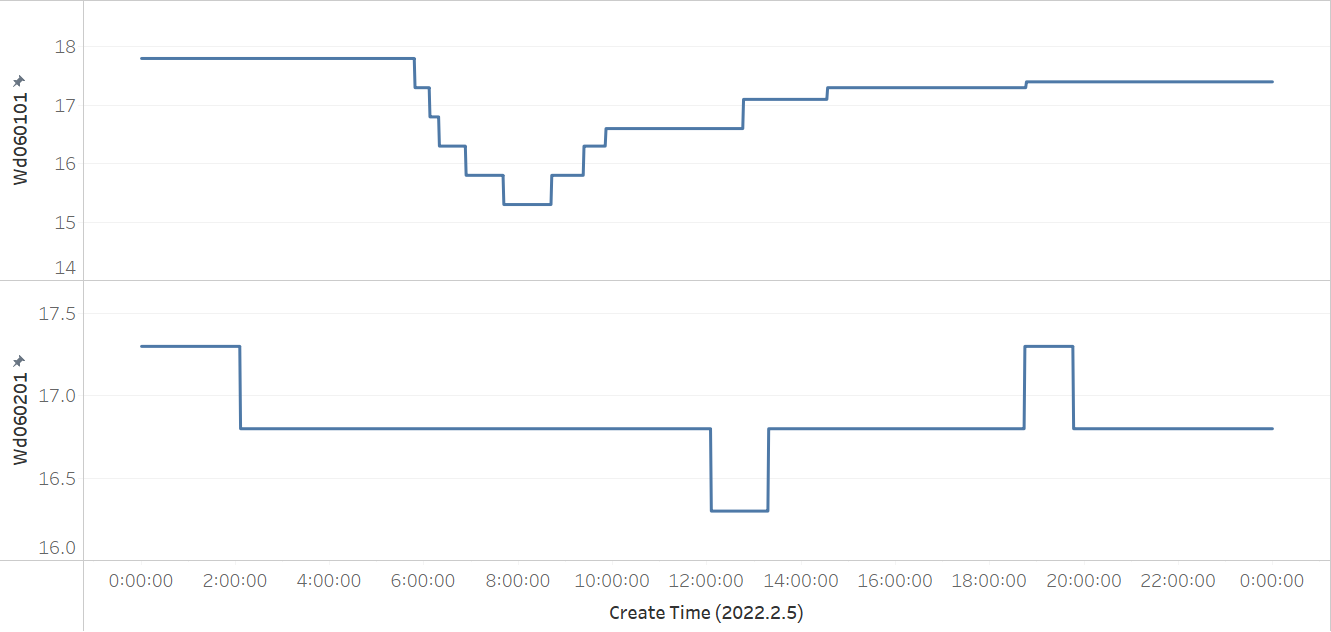


**Appendix 4: Wind Data on 5 Feb 2022**


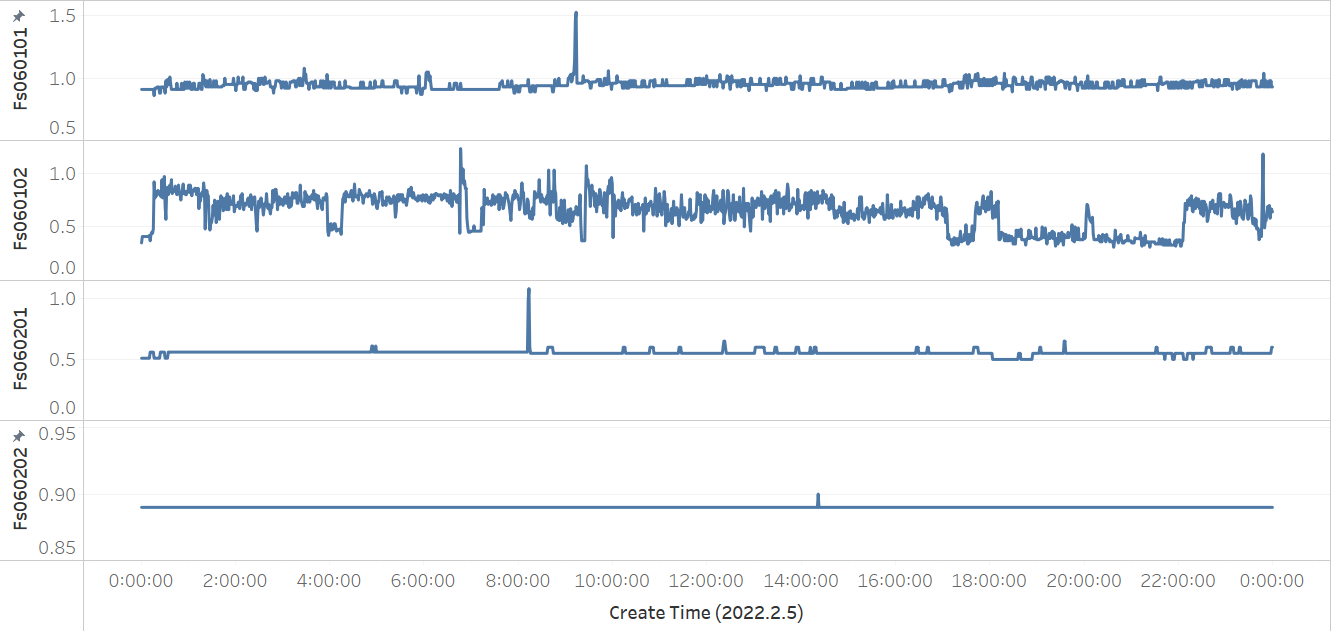


**Appendix 5: Gas Data on 6 Feb 2022**


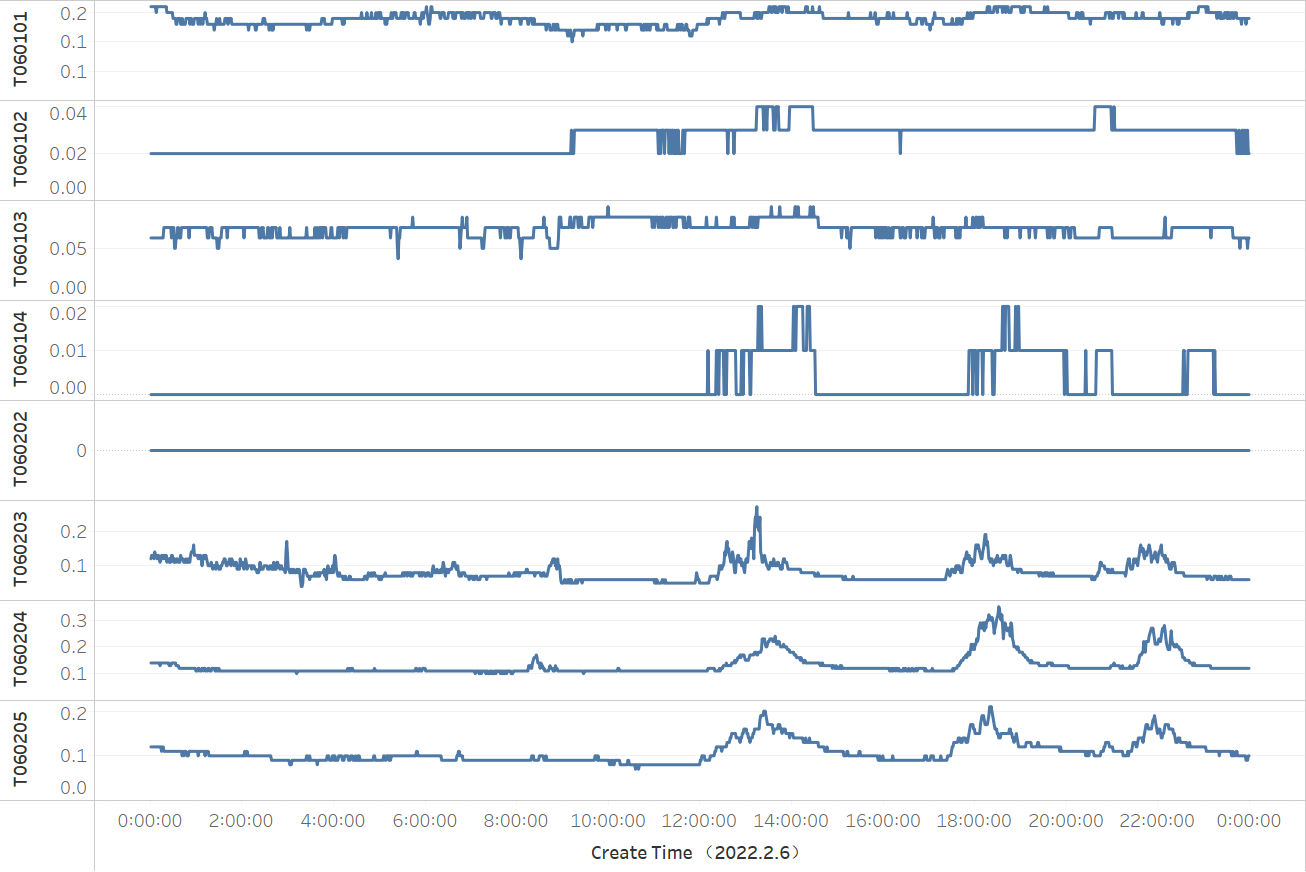


**Appendix 6: Temperature Data on 6 Feb 2022**


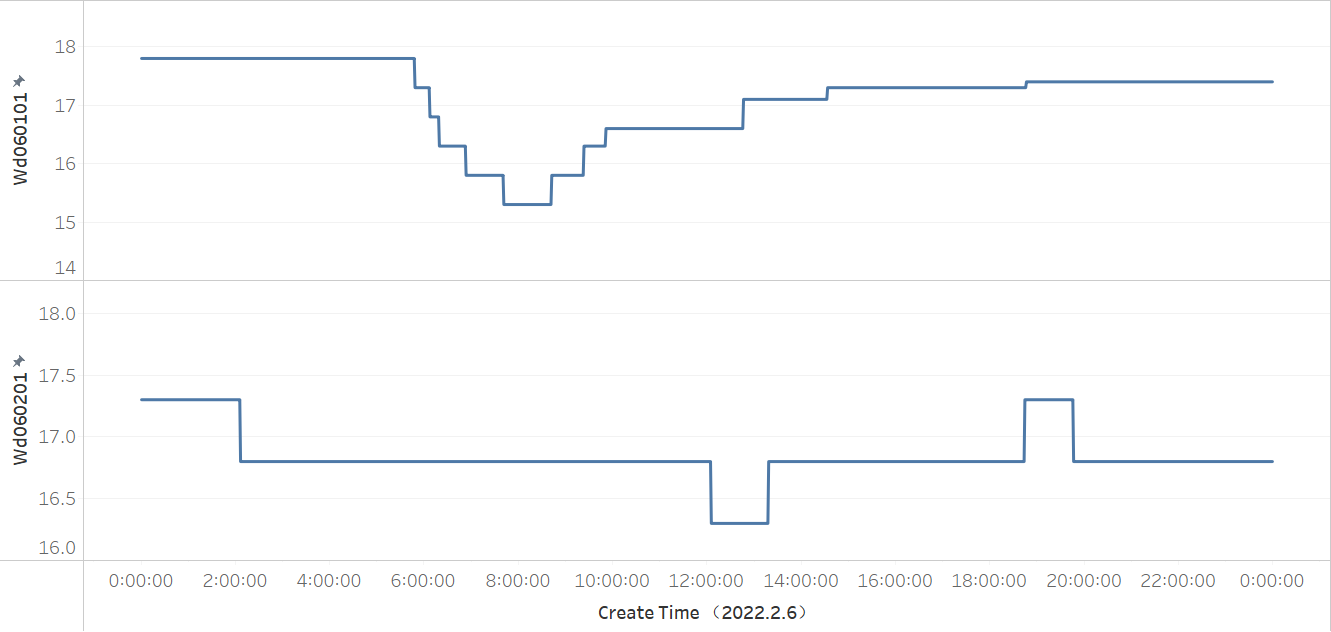


**Appendix 7: Wind Data on 6 Feb 2022**


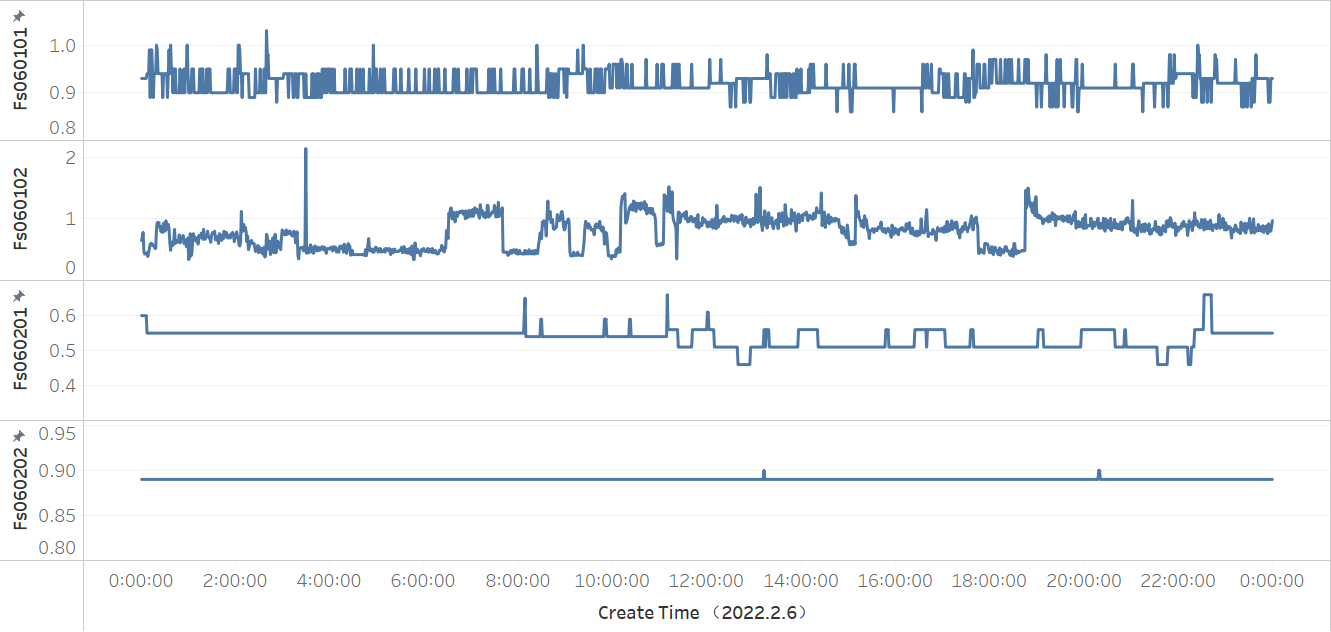


**Appendix 8: Pseudocode on the description of The System's Implementation**

~~
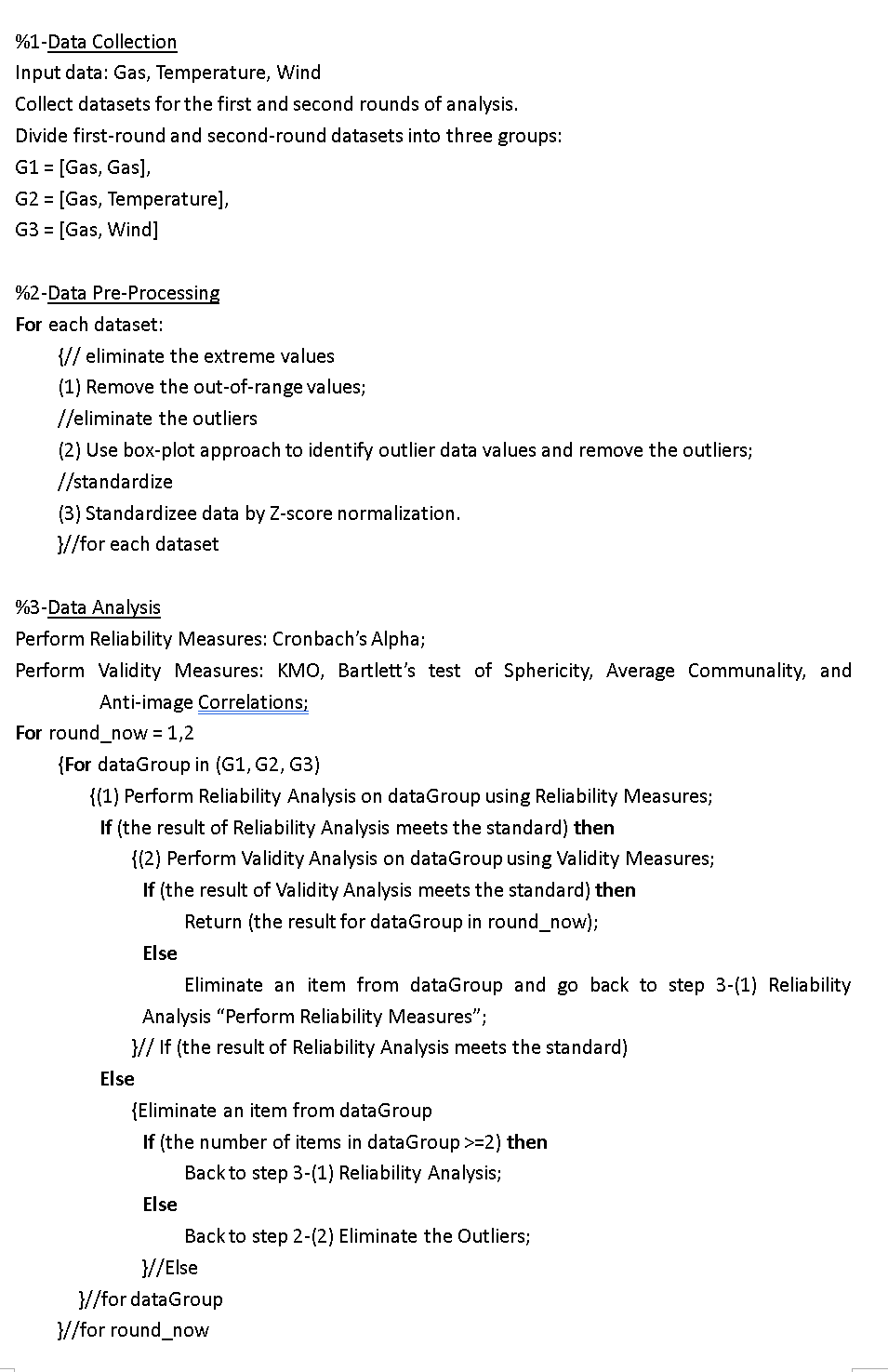
~~

~~
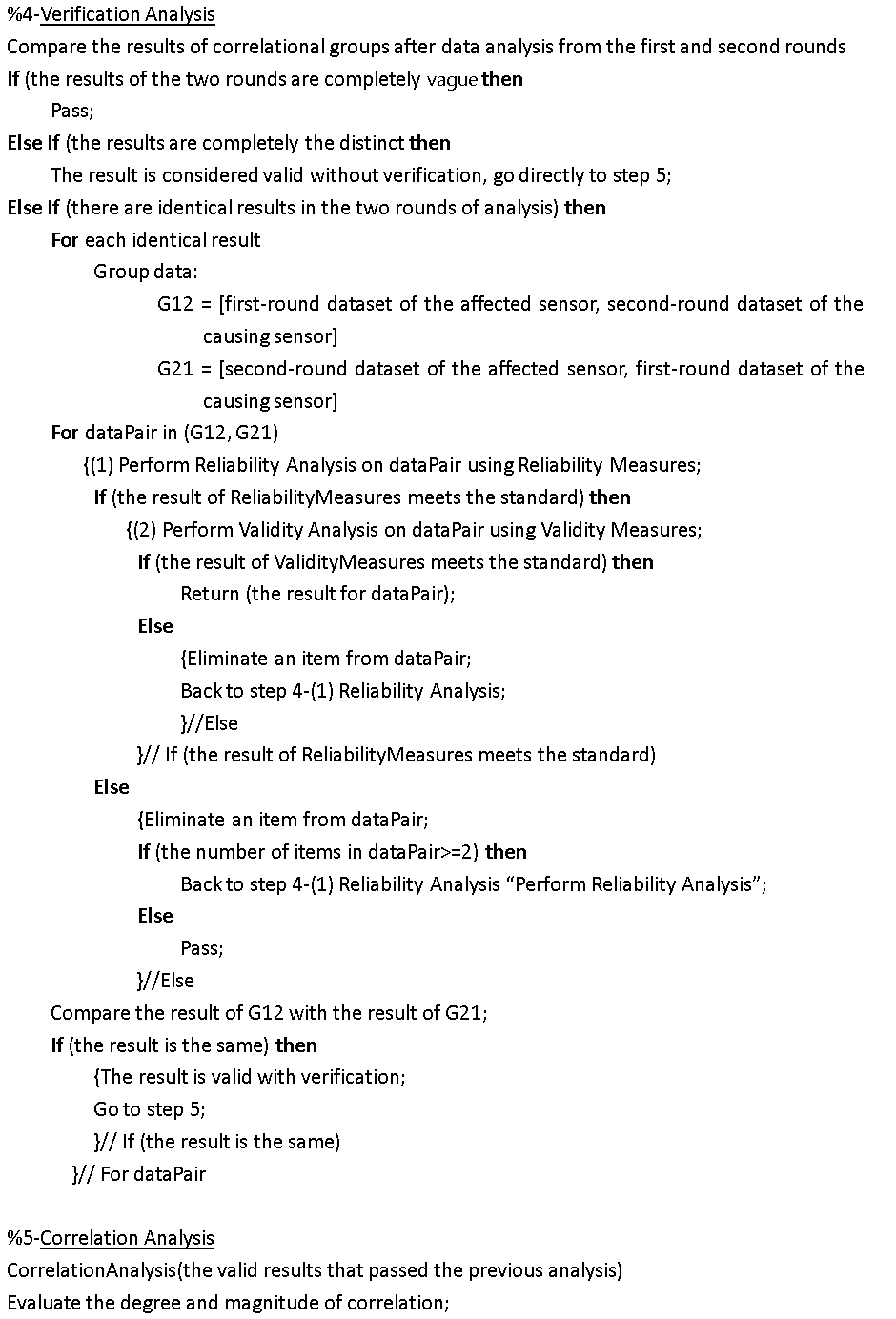
~~
